# Methicillin-resistant *Staphylococcus aureus* contamination in meat and meat products: a systematic review and meta-analysis

**DOI:** 10.3389/fmicb.2025.1636622

**Published:** 2025-07-15

**Authors:** Lili Xing, Mao Cheng, Shulei Wang, Jide Jiang, Ting Li, Xinyu Zhang, Jian Yang, Yunlong Tian, Wenjuan Liu

**Affiliations:** ^1^Bacteriological Disease Laboratory, Yantai Center for Disease Control and Prevention, Yantai, Shandong, China; ^2^Food Inspection and Testing Technology Teaching and Research Office, Yantai Engineering and Technology College, Yantai, Shandong, China; ^3^Department of Scientific Research and Education, Yantai Center for Disease Control and Prevention, Yantai, Shandong, China; ^4^Department of Biology, Gaoling Town Junior High School of Muping District, Yantai, Shandong, China; ^5^Pediatrics, Maternal and Child Health Center of Yantai Economic and Technological Development Zone, Yantai, Shandong, China

**Keywords:** meat and meat products, methicillin-resistant *Staphylococcus aureus* (MRSA), prevalence, systematic review, meta-analysis

## Abstract

**Background:**

Methicillin-resistant *Staphylococcus aureus* (MRSA) is distributed all over the world and can easily colonize food animals, which can be transmitted through the food chain, posing a threat to food safety and public health. This study aimed to elucidate the global prevalence of MRSA contamination in meat and meat products via systematic review and meta-analysis.

**Methods:**

A comprehensive retrieval was conducted in PubMed, Embase, Web of Science, and the Cochrane Library to identify eligible studies published up to December 10, 2024. Epidemiological data and study characteristics were extracted. Meta-analysis was made using a random-effects model in R software. Subgroup analyses were carried out by meat type, geographical region, and study period. Sensitivity analyses were launched to test the robustness of results, and the trim-and-fill method was applied to assess the potential impact of publication bias.

**Results:**

The pooled prevalence of MRSA contamination in meat and meat products was 3.72% (95% CI: 2.75–5.02%). The prevalence was 4.46% (95%CI: 2.82–6.98%) in raw poulty meat, 3.86% (95% CI: 2.58–5.74%) in raw livestock meat, and 2.84% (95%CI: 0.55–13.32%) in processed meat products. The Eastern Mediterranean region had the highest MRSA prevalence (9.13%; 95% CI: 4.28–18.44%), while North America reported the lowest (1.89%; 95% CI: 1.30–2.74%). Since 2015, the global prevalence of MRSA was 8.33% (95% CI: 5.29–12.86%). The adjusted pooled prevalence increased to 14.04% (95% CI: 10.38–18.73%) after applying the trim-and-fill method.

**Conclusion:**

The presence of MRSA in meat and meat products represents a public health concern. Enhanced surveillance efforts should prioritize raw livestock and poultry meat, particularly in the Eastern Mediterranean and Southeast Asia, to mitigate MRSA contamination in the food supply.

**Systematic review registration:**

CRD420251009933, https://www.crd.york.ac.uk/PROSPERO/view/CRD420251009933.

## 1 Introduction

Meat and meat products are essential to human diet, with their global consumption continuing to rise (Forcinio, 2022; Oleinikova et al., 2025). As reported by the Food and Agriculture Organization (FAO) of the United Nations, the production of meat has surged at an average annual growth rate of 1.5% over the past two decades, reaching over 360 million tons in 2022, with poultry and pork being the predominant types (FAO, 2024). Meat is rich in high-quality proteins, essential vitamins, and minerals, contributing significantly to human health (Geoffrey Tshifhiwa et al., 2021; Wagner et al., 2020). However, various stages of production, processing, storage, and distribution are vulnerable to contamination (Aydin et al., 2011; Kotzekidou, 2013), with microbial contamination posing a particularly serious threat to food safety.

The World Health Organization (WHO) reports that the global health burden of foodborne illnesses is comparable to that of malaria, tuberculosis, and HIV/AIDS, with bacterial pathogens responsible for approximately two-thirds of all cases (Havelaar et al., 2015). Animal-derived foods are major transmission vehicles (Grace, 2023; Hoffmann et al., 2017). *Staphylococcus aureus* (*S. aureus*), a leading cause of bacterial food poisoning, produces a range of heat-stable enterotoxins. It is widely distributed on the skin and mucosa of humans and animals, and meat’s rich protein content provides a favorable environment for its growth, potentially leading to acute gastrointestinal symptoms such as diarrhea and vomiting, and even death in severe cases.

More critically, in intensive livestock farming systems, the recurrent administration of antibiotics through feed additives or drinking water induces selective proliferation of antibiotic-resistant bacterial populations. Of particular concern is the emergence of methicillin-resistant *Staphylococcus aureus* (MRSA), which poses a threat to public health (Alqurashi et al., 2025; Junnila et al., 2020). MRSA exhibits an uncanny ability to colonize diverse anatomical niches, including nasal passages, skin surfaces, and gastrointestinal mucosa, across a spectrum of host species (Geenen et al., 2013; Nemati et al., 2009). Armed with the *mecA* gene or its *mecC* homolog, MRSA encodes penicillin-binding protein 2a (PBP2a), bestowing it with resilience against *β*-lactam antibiotics. This not only complicates treatment but also amplifies the risk of zoonotic transmission (Petinaki and Spiliopoulou, 2012). Once confined primarily to hospital-acquired infections, MRSA has now infiltrated community settings, livestock populations, and even the food supply chain, demonstrating clear attributes indicative of cross-regional transmission dynamics (Kourtis et al., 2019; Matuszewska et al., 2022). The extensive use of antimicrobials in animal husbandry has catalyzed a dramatic surge in the prevalence of MRSA among food-producing animals and their derived products. Alarmingly, foodborne MRSA strains often harbor multiple virulence factors and mobile genetic elements, which expedite the horizontal dissemination of antimicrobial resistance (AMR) genes across microbial populations. This trend exacerbates the dissemination of antimicrobial resistance and poses a significant threat to global public health security by perpetuating the cycle of antibiotic resistance (Ba et al., 2023).

As public demand for high-quality and safe food continues to escalate, the focus has increasingly shifted toward unraveling the epidemiology of MRSA in food-producing animals (Ribeiro et al., 2018). Studies have documented MRSA contamination in meat across various regions. For instance, in Aydın and İzmir, Turkey, the prevalence of MRSA in chicken samples was 2.0% (Kizanlik and Goksoy, 2024), while in traditional markets and supermarkets in Egypt, it reached 5.0% (Morshdy et al., 2023). In Bulgaria, retail pork samples had a prevalence of 4.7% (Gergana et al., 2024). Currently, a systematic global evaluation remains lacking regarding the spatiotemporal distribution patterns and category-specific variations in the prevalence of MRSA contamination across meat and meat products. As a critical method for integrating multi-source research data, meta-analysis enables the synthesis of multiple study outcomes, identifies heterogeneity among primary investigations, and elucidates sources of inter-study variation, thereby providing more robust evidence bases for quantitative microbial risk assessment.

This study employed systematic review and meta-analysis to quantify the global pooled prevalence of MRSA, establishing epidemiological evidence essential for scientifically evaluating foodborne disease burden assessments.

## 2 Materials and methods

This study followed the PRISMA guidelines (Page et al., 2021) and was prospectively registered in PROSPERO (CRD 420251009933).

### 2.1 Search strategy and literature screening

The search strategy was jointly developed by all authors. A systematic search was made in four databases: PubMed, Embase, Web of Science, and the Cochrane Library. The search spanned from each database’s inception to December 10, 2024. The core search terms included “meat” and “*Staphylococcus aureus*,” and the search was expanded using a combination of subject headings and free-text terms, with Boolean operators “OR” and “AND” to construct complex queries, for instance, “meat” OR “meat products” OR “sausage” AND “*Staphylococcus aureus*” OR “keflin *staphylococcus aureus*” OR “Micrococcus aureus” OR “Micrococcus pyogenes” OR “*Staphylococcus aureus* atcc 9801” OR “*Staphylococcus aureus* m strain” OR “*Staphylococcus aureus* smith strain” OR “Staphylococcus pyogenes aureus” OR “Staphylococcus pyogenes citreus.” The full search strategy and applied restrictions are provided in Supplementary Table S1.

All retrieved records were imported into EndNote 21 for deduplication. Two authors (Lili Xing and Mao Cheng) independently screened the titles, abstracts, and keywords. The remaining articles were then assessed through full-text reading to determine eligibility. Any discrepancies were settled through discussion with a third author (Jide Jiang).

### 2.2 Inclusion and exclusion criteria

Studies were included if they were original articles focusing exclusively on meat and meat products as research subjects. These studies must have clearly reported the original data necessary for calculating the prevalence of MRSA contamination, with sample data that could be independently extracted. Non-original research types, including reviews, commentaries, editorials, case reports, conference abstracts, and expert opinions, were excluded. Studies that focused on non-meat samples, lacked MRSA-related data, or presented data that were not extractable or insufficient data integrity resulting from unreported sample sizes were also excluded. Additionally, non-English publications and articles without full-text access were excluded.

### 2.3 Data extraction

Data were extracted using a pre-designed Microsoft Excel form. The extracted information included study characteristics (first author, year of publication, study year, sample size, and country), statistical data on meat and meat products (type of meat, sampling source, detection method), and relevant outcomes (number of MRSA-positive samples and total samples). For studies involving multiple types of meat and meat products, data for each category were recorded separately.

### 2.4 Quality assessment

The JBI critical appraisal tool^1^ was used to assess the quality of included studies. The JBI tool evaluates cohort studies based on the appropriateness of the sampling frame, sampling methods, sample size adequacy, description of study subjects, data analysis, identification of relevant conditions, measurement methods, statistical analysis, and response rate. Each item was scored as “Yes” = 1 and others = 0. A total score of ≥7 was considered high quality, 4–6 as moderate quality, and <4 as low quality.

### 2.5 Data analysis

Data analysis was conducted using R version 4.5.0 and the “meta” package. Selection among various transformation methods (e.g., arcsine, logit) was based on the normality of transformed data distributions. Following systematic comparison, PLOGIT-transformed data demonstrated the closest alignment with normal distribution patterns, thereby satisfying the fundamental assumptions required for random-effects model in meta-analytical procedures. Heterogeneity was assessed with Cochran’s Q test and the *I*^2^ statistic. A Q test *p* < 0.10 indicated significant heterogeneity, and *I^2^* values of 25, 50, and 75% were interpreted as low, moderate, and high heterogeneity, respectively (Higgins et al., 2003). To explore the sources of heterogeneity, subgroup analyses on MRSA contamination were conducted based on country, study period, and meat type. Meat products were categorized into raw livestock meat, raw poultry meat, processed meat products, and cooked meat. Processed meat products is defined as meat that has undergone transformation through salting, curing, fermentation, smoking, or other methods primarily to enhance flavor or extend shelf life (Humans IWGotEoCRt, 2018). Cooked meat refers to meat-based products produced through a series of processing steps such as ingredient selection, trimming, curing, seasoning, shaping, maturation, and packaging, with cooking being the defining characteristic. Studies were also stratified based on whether they were conducted before or after January 1, 2015. Additionally, regional variation in MRSA contamination was evaluated using both continental and WHO-defined regional groupings.^2^

Publication bias was assessed using funnel plots and Egger’s test. If publication bias was detected (asymmetric funnel plot or Egger’s test *p* < 0.10), trim-and-fill analysis was performed. Sensitivity analysis was conducted using leave-one-out analysis to assess the robustness of the pooled prevalence estimates. All analyses used a random-effects model with PLOGIT (logit-transformed proportion) estimation.

## 3 Results

### 3.1 Literature screening process and study characteristics

**Figure 1:**
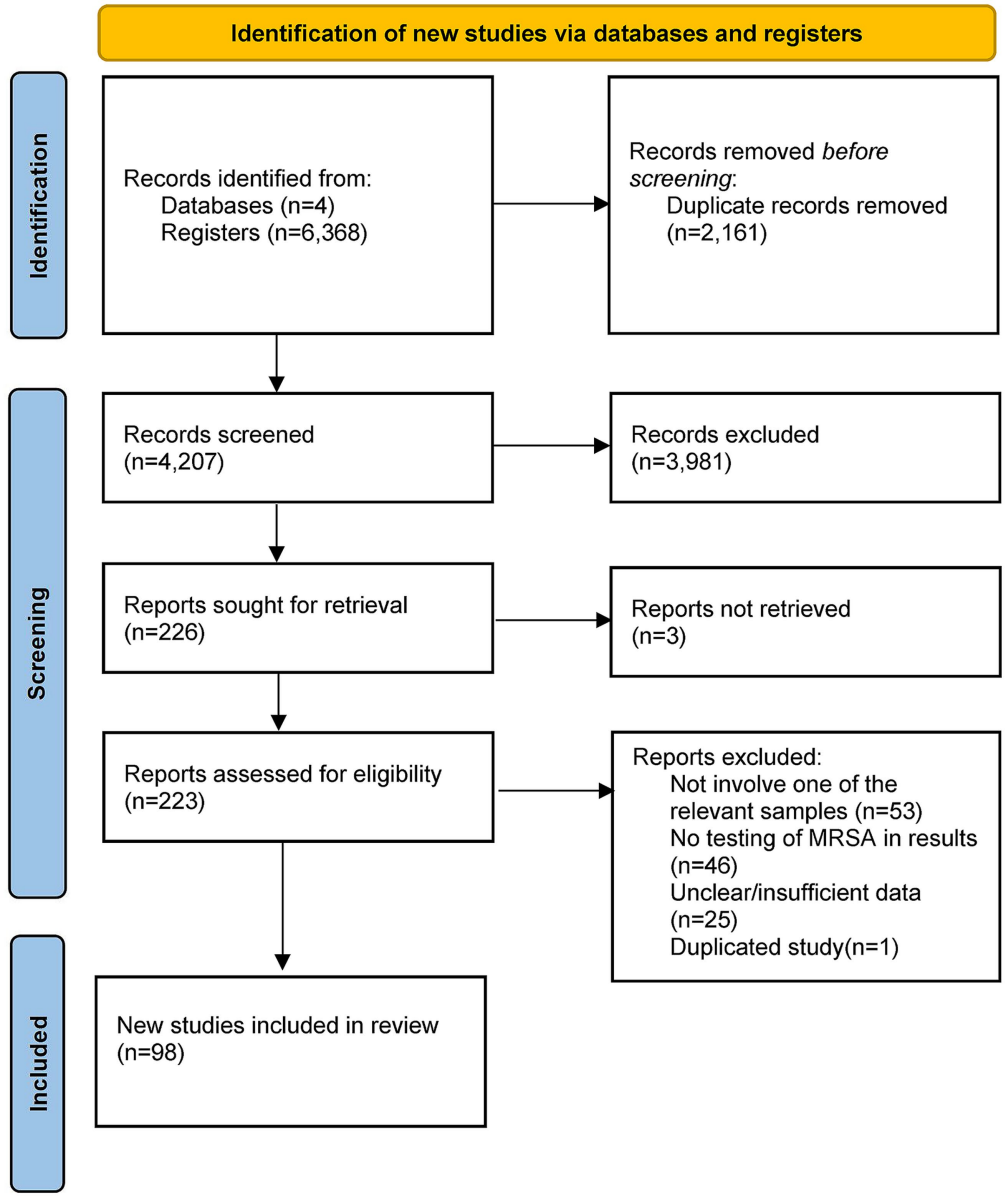
PRISMA literature screening flowchart.

A total of 6,368 articles were retrieved: 1,808 from PubMed, 1,974 from Embase, 2,581 from Web of Science, and 5 from the Cochrane Library. After removing 2,161 duplicates, 4,207 records remained. Following title, abstract, and keyword screening, 3,981 articles were excluded. After full-text screening, 98 articles (Supplementary File S1) were finally included in the meta-analysis. The selection process is illustrated in Figure 1.

The included studies were from 35 countries, including 12 from Europe, 13 from Asia, 5 from Africa, 3 from North America, and 2 from South America. A total of 34,103 samples were examined, covering raw livestock meat, raw poultry meat, processed meat products, and cooked meat. Supplementary Tables S2, S3 present the study characteristics and quality assessments using the JBI checklist. All included articles were assessed as high-quality.

### 3.2 Pooled prevalence of MRSA contamination in meat and meat products

The pooled prevalence of MRSA contamination in meat and meat products was 3.72% (95% CI: 2.75–5.02%). Substantial heterogeneity was observed (*I*^2^ = 95.1%, *p* < 0.001), as shown in Supplementary Figure S1. Sensitivity analysis via leave-one-out confirmed the robustness of this result (Supplementary Figure S2).

### 3.3 Regional differences in MRSA contamination

Studies represented all five continents, with most conducted in Asia (*n* = 56), Europe (*n* = 37), and North America (*n* = 27), followed by Africa (*n* = 17) and South America (*n* = 3). Subgroup analysis showed significant intergroup heterogeneity (*p* = 0.006). The prevalence was highest in South America (8.32, 95% CI: 0.74–52.51%), followed by Europe (5.47, 95% CI: 3.35–8.81%) and Asia (4.42, 95% CI: 2.60–7.41%). North America had the lowest prevalence at 1.89% (95% CI: 1.30–2.74%).

According to WHO regional classification, the Eastern Mediterranean region had the highest pooled prevalence of MRSA contamination (9.13, 95% CI: 4.28–18.44%), followed by the South-East Asia region (7.16, 95% CI: 1.28–31.40%). These two regions showed higher prevalence rates than others, with details as shown in Table 1.

**Table 1:** Prevalence of MRSA contamination in meat and meat products by region, meat type, and study period.

Subgroup	Number of studies	Sample size	Pooled prevalence (95% Cl)	*I* ^2^	*p*
Continent (Intergroup heterogeneity *p* = 0.006)					
Asia	56	15,103	0.0442 (0.0260 to 0.0741)	96.2%	<0.0001
Europe	37	5,532	0.0547 (0.0335 to 0.0881)	89.0%	<0.0001
Africa	17	1907	0.0295 (0.0108 to 0.0776)	89.2%	<0.0001
North America	27	11,407	0.0189 (0.0130 to 0.0274)	83.0%	<0.0001
South America	3	154	0.0832 (0.0074 to 0.5251)	42.4%	0.1761
WHO Region (Intergroup heterogeneity *p* = 0.005)					
Eastern Mediterranean	24	1955	0.0913 (0.0428 to 0.1844)	92.6%	<0.0001
Europe	46	6,537	0.0486 (0.0315 to 0.0742)	88.3%	<0.0001
Africa	5	916	0.0213 (0.0018 to 0.2050)	89.6%	<0.0001
Western Pacific	27	12,238	0.0210 (0.0114 to 0.0381)	95.9%	<0.0001
South-East Asia	8	896	0.0716 (0.0128 to 0.3140)	92.5%	<0.0001
Americas	30	11,561	0.0207 (0.0129 to 0.0331)	88.6%	<0.0001
Type of meat (intergroup heterogeneity *P* = 0.064)					
Processed meat products	7	589	0.0284 (0.0055 to 0.1332)	85.1%	<0.0001
Raw poutry meat	60	12,784	0.0446 (0.0282 to 0.0698)	95.0%	<0.0001
Raw livestock meat	68	20,239	0.0386 (0.0258 to 0.0574)	95.3%	<0.0001
Cooked meat	5	491	0.0010 (0.0001 to 0.0161)	0.00%	0.9951
Study period (intergroup heterogeneity *P* < 0.001)					
Post-2015	45	7,871	0.0833 (0.0529 to 0.1286)	94.7%	<0.0001
Unspecified	25	2,433	0.0192 (0.0084 to 0.0432)	90.9%	<0.0001
Pre-2015	70	23,799	0.0272 (0.0179 to 0.0409)	95.6%	<0.0001

### 3.4 Prevalence of MRSA contamination by meat type

Meta-analysis results by meat type are presented in Table 1. Most samples came from raw livestock meat (*n* = 20,239) with a pooled prevalence of 3.86% (95% CI: 2.58–5.74%). Raw poultry meat, the second most studied category (*n* = 12,784), showed the highest prevalence at 4.46% (95% CI: 2.82–6.98%). No MRSA was detected in cooked meat samples (*n* = 491). Intergroup heterogeneity was observed among the four meat categories (*p* = 0.064).

The pooled prevalence of MRSA contamination in the Eastern Mediterranean and Southeast Asia regions was 8.63% (95% CI: 4.28–16.63%), with specifically 10.50% (95% CI: 4.69–21.86%) in raw poultry meat and 7.52% (95% CI:1.85–25.99%) in raw livestock meat.

### 3.5 Prevalence of MRSA contamination over time

The prevalence of MRSA contamination in meat and meat products differs significantly across different time periods. Among studies with clearly defined temporal data, the prevalence was 2.72% from 2003 to 2014 and increased to 8.33% from 2015 to 2024. Detailed information regarding the 95% CI and heterogeneity is provided in Table 1. Meta-regression revealed a temporal increase in the prevalence of MRSA contamination (*p* = 0.0047), though this trend was not observed in raw poultry meat (*p* = 0.0933) (Table 2).

**Table 2:** Univariable meta-regression analysis for prevalence of MRSA contamination as dependent variable accounting for year.

Group	Studies	Regression coefficient (95% CI)	*p*-value
Meat and meat products	112	0.1049 (0.0322 to 0.1776)	0.0047
Raw poultry meat	52	0.1029(−0.0173 to 0.2231)	0.0933
Raw livestock meat	55	0.1193 (0.0307 to 0.2080)	0.0083

### 3.6 Prevalence of *mecA* and *mecC* genes

A total of 129 studies investigated the prevalence of *mecA* and *mecC* genes in MRSA isolates from meat and meat products. Among these, 106 studies reported a pooled prevalence rate of *mecA*-positive MRSA at 5.51% (95% CI: 4.09–7.39%), with substantial heterogeneity (*I*^2^ = 96.0%, *p* < 0.0001). In contrast, 26 studies documented a markedly lower prevalence of *mecC*-carrying MRSA at 0.07% (95% CI: 0.03–0.15%), demonstrating negligible heterogeneity (*I*^2^ = 0.0%, *p* = 1.0000) (Supplementary Figures S3, S4).

### 3.7 Publication bias

The funnel plot is shown in Supplementary Figure S5. Egger’s test revealed significant publication bias (*p* < 0.0001). After applying the trim-and-fill method, the adjusted prevalence was 14.04% (95% CI: 10.38–18.73%). The adjusted funnel plot is shown in Supplementary Figure S6.

## 4 Discussion

The systematic review and meta-analysis integrated 34,103 samples from 98 studies across 35 countries, comprehensively quantifying the global prevalence of MRSA contamination in meat and meat products up to December 2024. The findings elucidate spatiotemporal distribution patterns and heterogeneity sources. The pooled prevalence of MRSA was estimated at 3.72% (95% CI: 2.75–5.02%), with significant heterogeneity observed across studies (*I^2^* = 95.1%, *p* < 0.001). This prevalence is lower than that reported by Ribeiro et al. (2018) in poultry meat (8%) but similar to the findings of Ou et al. (2017) on raw meat (3.2%), indicating the widespread dissemination of MRSA along the food chain. After applying the trim-and-fill method to adjust for potential publication bias, the prevalence rose to 14.04% (95% CI: 10.38–18.73%). This dramatic increase suggests that the actual MRSA contamination levels may substantially exceed published estimates due to publication bias favoring negative results, unaccounted cross-contamination risks during retail distribution, and methodological limitations of conventional culture-based detection missing viable-but-non-culturable MRSA states. Enhanced surveillance protocols, advanced detection technologies, and improved data reporting mechanisms are urgently needed for accurate MRSA risk assessment and control.

Notably, the Eastern Mediterranean (9.13%) and Southeast Asia (7.16%) exhibited considerably higher MRSA prevalence (8.63%) than other regions, whereas North America showed the lowest prevalence (1.89%). These regional disparities may be attributable to various factors. The Eastern Mediterranean and South-East Asian regions, both located in tropical zones, provide warm and humid climates favorable for *S. aureus* proliferation, thereby increasing the risk of contamination during meat processing and storage. Predominant intensive farming systems in these regions accelerate pathogen transmission through high-density poultry/livestock confinement (Sykes et al., 2023; Chapot et al., 2024; Rizzo et al., 2024). Biosecurity protocols are inadequately implemented during frequent animal trade activities (Guo et al., 2018). Regulatory deficiencies include absence of mandatory antibiotic residue limits in feed additives in Southeast Asia, and weak antibiotic procurement traceability systems in Eastern Mediterranean, resulting in systematic antibiotic misuse as growth promoters and prophylactic agents. Such practices drive unregulated human-animal exposure that serves as a driver for MRSA evolution and dissemination (Adesokan et al., 2015; Lakhundi and Zhang, 2018; Malik et al., 2023). As most investigated nations are low-and middle-income countries, additional causal factors likely involve suboptimal sanitation infrastructure and cross-contamination during slaughtering and meat processing (Abolghait et al., 2020; Verhegghe et al., 2015). Moreover, weaknesses in cold chain logistics and high ambient temperatures during retail storage may further facilitate bacterial persistence. By contrast, the low prevalence observed in North America aligns with two prior meta-analyses (Ribeiro et al., 2018; Ou et al., 2017), potentially reflecting stringent hygiene regulations, the effective implementation of Hazard Analysis and Critical Control Points (HACCP) systems (Hwang et al., 2022), and standardized antimicrobial stewardship practices in livestock farming (Federal Register, 2012; Government of Canada, 2015). However, the findings for South America should be interpreted with caution due to the limited number of studies (*n* = 3) available for this region.

The prevalence of MRSA contamination varies across different types of meat and meat products. Raw poultry meat exhibited the highest pooled prevalence (4.46%), followed by raw livestock meat (3.86%). This observation may be attributed to the high colonization potential of MRSA on the gastrointestinal tract and skin surfaces of poultry and livestock (Peeters et al., 2015; Rinsky et al., 2013). The intensive production systems commonly used in animal husbandry may increase the risk of cross-contamination (Guo et al., 2018), and lapses in hygienic practices during slaughter further exacerbate the spread of contamination. Inadequate control of environmental conditions, such as temperature and humidity during transportation and retailing also creates favorable conditions for bacterial proliferation. Moreover, the high protein and fat content of raw meat provides a nutrient-rich environment conducive to bacterial growth (Zhou et al., 2010). In supermarkets or butcheries, meat packaged in polystyrene trays with polyethylene film requires additional handling procedures that may increase the risk of MRSA contamination (Gombas et al., 2017). Moreover, sampled meat packaging demonstrated breached integrity at collection, a critical factor exacerbating cross-contamination potential (Xin et al., 2013), though quantitative comparative data on MRSA contamination across packaging modalities remain lacking. Processed meat products showed a relatively lower prevalence (2.84%), which may be due to osmotic pressure changes during marination or seasoning that inhibit bacterial growth. However, if storage conditions are suboptimal, such products can still serve as potential carriers for toxin accumulation. The absence of MRSA in cooked meat likely reflects synergistic effects of high-temperature sterilization protocols and preferential sourcing from retail establishments with standardized hygiene management (326/491, 66.4%). However, these conclusions derive from only five studies with limited sample size (*n* = 491), potentially compromising result generalizability.

The contamination rate of MRSA in meat and meat products has exhibited a progressively increasing trend over the years. When stratified by study period, the prevalence of MRSA contamination in meat and meat products was higher from 2015 to 2024 (8.33%) than from 2003 to 2014 (2.72%). This increase may be related to the extensive use of antimicrobial agents in livestock production in recent years, which has promoted the emergence of antimicrobial-resistant bacteria. Furthermore, advancements in detection technologies, particularly the increasing sensitivity of molecular diagnostics and the widespread application of whole genome sequencing, have enabled more accurate identification of previously undetected strains. Increased public awareness of food safety issues has also led to a surge in relevant studies, which has contributed to uncovering the severity of MRSA contamination.

The prevalence of *mecA*-carrying MRSA in meat and meat products was estimated at 5.51%, with significant heterogeneity (*I*^2^ = 96.0%). This elevated heterogeneity may be attributed to differences in study designs, including specimen sources, methodological differences, and geographical variations. While horizontal gene transfer is a primary mechanism for *mecA* dissemination, antimicrobial selection pressure and host immune responses in meat production environments may constrain its epidemiological persistence. In contrast, the prevalence of *mecC*-carrying MRSA remained markedly low at 0.07%, indicative of limited ecological transmission efficiency in these settings. Notably, phenotypic misclassification of *mecC*-positive strains as methicillin-susceptible *Staphylococcus aureus* (MSSA) during routine susceptibility testing may contribute to underreporting (Lakhundi and Zhang, 2018; Paterson et al., 2014). Despite its low prevalence, *mecC*-carrying MRSA warrants vigilance due to its expanded resistance profiles and potential for immune evasion mechanisms.

Publication bias analysis revealed an asymmetric funnel plot and a statistically significant Egger’s test (*p* < 0.0001), suggesting possible bias in the published literature. This bias may stem from the tendency for studies with positive findings to be more likely to be published, while those with negative results remain unpublished or overlooked. Variations in sample size, detection methods, and other methodological factors across studies could also introduce heterogeneity. Although subgroup analyses were performed to address these differences, residual confounding may still influence the pooled estimates. Nevertheless, sensitivity analyses demonstrated the robustness of the pooled prevalence, with prevalence estimates ranging only from 5.10% (95% CI: 4.87–5.34%) to 5.94% (95% CI: 5.68–6.21%) after sequential exclusion of individual studies, thereby reinforcing the reliability of the findings.

Globally, bacterial contamination of meat is on the rise (Yu et al., 2021), frequently resulting in foodborne illnesses. MRSA has been detected at various stages of meat processing and in both live food animals (Porrero et al., 2012; Mama et al., 2019) and final retail products. Most contamination in food samples occurs in raw meat products. High-temperature cooking is effective in reducing *S. aureus* contamination in raw meats. Good food handling practices, including proper cooking and hygienic processing, are critical for minimizing the incidence of foodborne diseases (Yasin et al., 2012). These research findings provide evidence-based guidance for regulatory agencies to implement precision quality control interventions, thereby enhancing meat product safety for consumers. For high-prevalence geographical regions and contamination-prone commodities (specifically raw poultry/livestock meats), it is recommended to implement risk-based surveillance strategies, full-chain hygiene protocols encompassing husbandry, slaughtering, processing, transportation, and retail distribution; agricultural practice reformulation emphasizing antibiotic stewardship programs; and mandatory abattoir certification systems. There is a potential link between MRSA contamination rates and the burden of foodborne diseases. Understanding the prevalence of MRSA in meat and meat products can assist clinicians in making informed decisions on antimicrobial therapy and help curb the worsening of antimicrobial resistance due to inappropriate antimicrobial use. *S. aureus* toxins are major virulence factors responsible for food poisoning in both humans and animals (Zhu et al., 2023). MRSA strains carrying virulence genes such as PVL (Panton-Valentine leukocidin) may contribute to more severe clinical infections. While mechanistically possible, rigorous epidemiological investigation remains imperative to delineate the infection risks associated with direct meat contact or foodborne exposure routes.

## 5 Conclusion

In summary, the overall pooled prevalence of MRSA contamination in meat and meat products stood at 3.72% (95% CI: 2.75–5.02%), though this figure may be an underestimate. Following trim-and-fill analysis, the adjusted prevalence climbed to 14.04% (95% CI: 10.38–18.73%). In recent years, there has been a notable rise in the proportion of MRSA-contaminated meat, with detections increasingly reported in raw poultry meat, raw livestock meat, and processed meat products. The highest prevalence of MRSA was observed in the Eastern Mediterranean and Southeast Asian regions, with substantial geographical variability across continents. These findings underscore that MRSA contamination in meat poses a public health threat to consumers. Hygienic concerns regarding meat products must be integrated into strategies aimed at preventing and controlling contamination risks. Robust and targeted interventions are imperative to mitigate the risk of meat contamination and to prevent zoonotic transmission of MRSA to humans.

## Data Availability

The original contributions presented in the study are included in the article/Supplementary material, further inquiries can be directed to the corresponding authors.

## Footnotes

^1^ https://jbi.global/critical-appraisal-tools

^2^ https://www.who.int/about/who-we-are/regional-offices
