# Supplementary material for: Methicillin-resistant Staphylococcus aureus contamination in meat and meat products: a systematic review and meta-analysis
Source: Front Microbiol. 2025 Jul 15;16:1636622. doi: 10.3389/fmicb.2025.1636622 (PMC12303875; doi:10.3389/fmicb.2025.1636622)
Supplement: Supplementary file 1 [file Table_1.DOCX]

**Table S1** The full search strategy and applied restrictions.

| Pubmed | | |
| --- | --- | --- |
| # | Query | Results |
| 1 | Meat | 83510 |
| 2 | "meat" OR "Meats" OR "meat products" OR "sausage" | 77944 |
| 3 | Staphylococcus aureus | 95235 |
| 4 | "Staphylococcus aureus" OR "keflin staphylococcus aureus" OR "Micrococcus aureus" OR "Micrococcus pyogenes" OR "Staphylococcus aureus atcc 9801" OR "Staphylococcus aureus m strain" OR "Staphylococcus aureus smith strain" OR "Staphylococcus pyogenes aureus" OR "Staphylococcus pyogenes citreus" | 136196 |
| 5 | (#1 OR #2) AND (#3 or #4) | 1808 |

| Embase | | |
| --- | --- | --- |
| # | Query | Results |
| 1 | ‘Meat’/exp | 73062 |
| 2 | ('meat' OR 'Meats' OR 'meat products' OR 'sausage'):ti,ab,kw | 83683 |
| 3 | ‘Staphylococcus aureus’/exp | 235548 |
| 4 | ('Staphylococcus aureus' OR 'keflin staphylococcus aureus' OR 'Micrococcus aureus' OR 'Micrococcus pyogenes' OR 'Staphylococcus aureus atcc 9801' OR 'Staphylococcus aureus m strain' OR 'Staphylococcus aureus smith strain' OR 'Staphylococcus pyogenes aureus' OR 'Staphylococcus pyogenes citreus'):ti,ab,kw | 167773 |
| 5 | (#1 OR #2) AND (#3 or #4) | 1974 |

| Cochrane Library | | |
| --- | --- | --- |
| # | Query | Results |
| 1 | Meat | 1093 |
| 2 | ('meat' OR 'Meats' OR 'meat products' OR 'sausage'):ti,ab,kw | 3127 |
| 3 | Staphylococcus aureus | 1195 |
| 4 | ('Staphylococcus aureus' OR 'keflin staphylococcus aureus' OR 'Micrococcus aureus' OR 'Micrococcus pyogenes' OR 'Staphylococcus aureus atcc 9801' OR 'Staphylococcus aureus m strain' OR 'Staphylococcus aureus smith strain' OR 'Staphylococcus pyogenes aureus' OR 'Staphylococcus pyogenes citreus'):ti,ab,kw | 4174 |
| 5 | (#1 OR #2) AND (#3 or #4) | 5 |

| Web of Science | |
| --- | --- |
| Query | Results |
| "meat" OR "Meats" OR "meat products" OR "sausage" | 142491 |
| "Staphylococcus aureus" OR "keflin staphylococcus aureus" OR "Micrococcus aureus" OR "Micrococcus pyogenes" OR "Staphylococcus aureus atcc 9801" OR "Staphylococcus aureus m strain" OR "Staphylococcus aureus smith strain" OR "Staphylococcus pyogenes aureus" OR "Staphylococcus pyogenes citreus" | 169071 |
| #1 AND #2 | 2581 |

**Table S2** Basic characteristics of included studies.

| **No.** | **The surname of the first author** | **Year of publication** | **Country** | **Meat​** | **Sample source** | **Study period** | **Test methods​** | **Sample size** | **Number of MRSA contaminations detected** |
| --- | --- | --- | --- | --- | --- | --- | --- | --- | --- |
| 1 | Sheet | 2024 | Iraq | Basturma | Local shops in Mosul | April to June 2023 | Traditional microbiology techniques, PCR technique | 45 | 3 |
| 2 | Kizanlik | 2024 | Turkey | Chicken | Traditional markets, supermarkets and butcher shops in Aydin and Izmir | NA | Microbiological methods (ISO standards), multiplex PCR, VITEK2 | 100 | 2 |
| 3 | Gonzalez-Fandos | 2024 | Spain | Horsemeat | Two supermarkets in Logroño, Spain | June to September 2020 | ChromID MRSA Agar medium, Kirby-Bauer | 19 | 0 |
| 4 | Eguizábal | 2024 | Spain | Chicken | 16 supermarkets and 15 local butchers in La Rioja, Spain | July to December 2020, February to March 2023 | ORSAB culture medium, MALDI-TOF mass spectrometry identification | 60 | 1 |
| 5 | Guedes | 2024 | Spain | Rabbit meat | Traditional stores, supermarkets, hypermarkets | NA | ChromID MRSA agar culture medium, MALDI-TOF MS mass spectrometry identification (CLSI standard to confirm drug resistance) | 49 | 1 |
| 6 | Sheet | 2023 | Iraq | Veal | Butcher's shop in Mosul | September 2021 to January 2022 | PCR for detecting mecA gene | 50 | 24 |
| 7 | Naeim | 2023 | Egypt | Chicken | Local markets in different regions | August to November 2021 | ORSAB medium, PCR technique for detecting mecA and mecC genes | 25 | 3 |
|  | Naeim | 2023 | Egypt | Beef | Local markets in different regions | August to November 2021 | ORSAB medium, PCR technique for detecting mecA and mecC genes | 24 | 1 |
| 8 | Morshdy | 2023 | Egypt | Chicken | Traditional markets and supermarkets | September to November 2021 | PCR technique for detecting mecA gene | 200 | 10 |
| 9 | Martinez-Laorden | 2023 | Spain | Turkey | Retail Market | January 2020 to January 2021 | chromID MRSA agar medium, diffusion agar method (cefoxitin) | 51 | 4 |
| 10 | Igbinosa | 2023 | Nigeria | Poultry | Frozen chicken in the retail market | June 2018 to April 2019 | Standard culture method, PCR detection of nuc gene | 368 | 110 |
| 11 | Dorjgochoo | 2023 | Mongolia | Raw beef | 4 retail markets | June to December 2021 | Phenotypic Kirby-Bauer method (oxacillin resistance screening), PCR for mecA detection verification | 100 | 6 |
| 12 | Zhu | 2022 | China | Pork | Wholesale and retail markets | 2016 to 2017 | Phenotypic MIC method (oxacillin resistance), PCR for detecting mecA/mecC genes | 3067 | 19 |
| 13 | Thwala | 2022 | South Africa | Ready-to-eat beef | Retail stores and slaughterhouses | NA | Susceptibility testing, PCR for detecting mecA/mecC genes | 68 | 0 |
|  | Thwala | 2022 | South Africa | Raw processed beef | Retail stores and slaughterhouses | NA | Susceptibility testing, PCR for detecting mecA/mecC genes | 110 | 0 |
|  | Thwala | 2022 | South Africa | Beef (whole, unprocessed) | Retail stores and slaughterhouses | NA | Susceptibility testing, PCR for detecting mecA/mecC genes | 222 | 2 |
| 14 | Osada | 2022 | Japan | Chicken | Retail stores | May to September 2021 | PCR commercial kit for detecting mecA gene using broth microdilution method | 93 | 0 |
|  | Osada | 2022 | Japan | Pork, beef /pork mix | Retail stores | May to September 2021 | PCR commercial kit for detecting mecA gene using broth microdilution method | 53 | 0 |
| 15 | Komodromos | 2022 | Greece | Raw meat (beef, pork, lamb), meat products (ground and non-ground) | Two meat processing plants | NA | Cefoxitin (FOX) resistance test, PCR for detecting mecA/mecC genes | 61 | 1 |
| 16 | Guran | 2022 | Turkey | Beef | Retail markets (supermarkets and butchers) | September 2018 to January 2019 | Primary enrichment, selective culture, cefoxitin Kirby-Bauer, PCR for detecting nuc and mecA genes | 100 | 3 |
|  | Guran | 2022 | Turkey | Chicken, turkey, duck | Retail markets (supermarkets and butchers) | September 2018 to January 2019 | Primary enrichment, selective culture, cefoxitin disk diffusion, PCR for detecting nuc and mecA genes | 225 | 1 |
| 17 | Telli | 2021 | Turkey | Beef retail | Retail stores | February 2018 to March 2019 | The minimum inhibitory concentration (MIC) of oxacillin determined by microdilution method, and PCR for detecting mecA gene | 20 | 1 |
|  | Telli | 2021 | Turkey | Chicken retail | Retail stores | February 2018 to March 2019 | The minimum inhibitory concentration (MIC) of oxacillin determined by microdilution method, and PCR for detecting mecA gene | 20 | 1 |
| 18 | Tegegne | 2021 | Czech Republic | Poultry (turkey, chicken) | Retail market (supermarkets in different countries) | 2017 to 2018 | PCR for detection of mecA gene and S. aureus specific fragment Sa442, confirmed by MALDI-TOF/MS and drug sensitivity test | 33 | 12 |
|  | Tegegne | 2021 | Czech Republic | Beef, pork, rabbit meat, mixed minced meat (beef and pork) | Retail market (supermarkets in different countries) | 2017 to 2018 | PCR for detection of mecA gene and S. aureus specific fragment Sa442, confirmed by MALDI-TOF/MS and drug sensitivity test | 32 | 11 |
| 19 | Tanomsridachchai | 2021 | Thailand | Pork | Retail market | 2017 to 2018 | PCR for detecting mecA gene and S. aureus specific fragment Sa442, confirmed by MALDI-TOF/MS and drug sensitivity test | 116 | 52 |
| 20 | Parvin | 2021 | Bangladesh | Frozen chicken | Five metropolitan superstores | April to December 2019 | PCR for detecting nuc and mecA genes, Kirby-Bauer method for drug susceptibility testing | 113 | 23 |
| 21 | Martínez-Vázquez | 2021 | Mexico | Beef, pork | 55 retail stores and supermarkets in 11 cities | August 2013–March 2014 | PCR for detecting nuc and mecA genes, agar diffusion method for drug sensitivity testing | 106 | 3 |
| 22 | Sankomkai | 2020 | Thailand | Fermented pork sausage | Local retail market | NA | Kirby-Bauer method (detects resistance to beta-lactam antibiotics, including penicillin and cefoxitin) | 60 | 0 |
| 23 | Sadiq | 2020 | Pakistan | Beef, lamb | Slaughterhouses and butcher shops by region | 2018 to 2019 | Cefoxitin Kirby-Bauer method, PCR for detecting mecA | 70 | 40 |
|  | Sadiq | 2020 | Pakistan | Chicken | Slaughterhouses and butcher shops by region | 2018 to 2019 | Cefoxitin Kirby-Bauer method, PCR for detecting mecA | 30 | 23 |
| 24 | Ogundipe | 2020 | Nigeria | Chicken (dressed chicken, frozen chicken) | Live poultry market | December 2016–April 2017 | Selective culture, PCR detection of mecA gene | 148 | 30 |
| 25 | Kim | 2020 | Korea | Beef, pork | Domestic and imported meat | 2013 to 2018 | Broth microdilution method for drug sensitivity testing, PCR for detection of mecA gene confirmation | 3047 | 23 |
|  | Kim | 2020 | Korea | Chicken | Domestic and imported meat | 2013 to 2018 | Broth microdilution method for drug sensitivity testing, PCR for detection of mecA gene confirmation | 1217 | 6 |
| 26 | Bouchami | 2020 | Portugal | Pork | Domestic (Portugal) and imported (mainly from Spain) | 2016 (Summer and Winter) | CHROMagar MRSA selective medium, PCR for detection of mecA gene confirmation | 27 | 6 |
| 27 | Bernier-Lachance | 2020 | Canada | Chicken | 43 retail stores (Monterégie area) | June 2013 to November 2013 | MRSA agar, PCR | 309 | 4 |
| 28 | Basanisi | 2020 | Italy | Beef, pork, horsemeat | Retail market (Southern Italy) | June 2016 to June 2018 | Multiplex PCR for detecting nuc and mecA/mecC genes | 350 | 11 |
|  | Basanisi | 2020 | Italy | Chicken | Retail market (Southern Italy) | June 2016 to June 2018 | Multiplex PCR detection of nuc and mecA/mecC genes | 150 | 1 |
| 29 | Sivakumar | 2019 | India | Raw chicken | Street vendors (vendors in Delhi and Bareilly), different suppliers | September 2015–May 2016 | Culture method combined with biochemical identification, PCR for detecting nuc and mecA genes, MeReSa agar screening, drug sensitivity testing (Kirby-Bauer method) | 39 | 2 |
| 30 | Naas | 2019 | Libya | Red meat | Retail market | NA | Baird-Parker medium culture, PCR and 16S rDNA sequencing | 44 | 0 |
|  | Naas | 2019 | Libya | Chicken | Retail market | NA | Baird-Parker medium culture, PCR and 16S rDNA sequencing | 10 | 0 |
| 31 | El-Ghareeb | 2019 | Saudi Arabia | Camel meat | Retail market (Al-Hasa Area) | 5 months | Cefoxitin resistance Kirby-Bauer method, PCR for detection of mecA gene confirmation | 187 | 3 |
| 32 | Kim | 2018 | Korea | Chicken | Four retail markets of seven different integrated broiler producers | 2016 | PCR for detection of mecA gene and agar dilution method recommended by CLSI | 200 | 4 |
| 33 | Thapaliya | 2017 | America | Chicken, ground turkey | Commercial meat (both conventional and antibiotic-free meat) from eight retail stores in Iowa | January to December 2012 | PCR for detection of mecA gene and PVL gene, CHROMagar MRSA selective medium | 1033 | 14 |
|  | Thapaliya | 2017 | America | Beef, pork | Commercial meat (both conventional and antibiotic-free meat) from eight retail stores in Iowa | January to December 2012 | PCR For detection of mecA gene and PVL gene, CHROMagar MRSA selective medium | 2211 | 27 |
| 34 | Osman | 2017 | Egypt | Imported frozen beef | Egyptian market | NA | PCR for detection of mecA gene, phenotypic drug sensitivity test | 100 | 0 |
| 35 | Arslan | 2017 | Turkey | Ground beef | Public bazaar, supermarket and butcher shop in Bolu, Türkiye | October 2011–December 2012 | PCR For detection of mecA gene, Kirby-Bauer method | 40 | 0 |
| 36 | Zogg | 2016 | Switzerland | Raw chicken | Switzerland (36) and imports (44 from Argentina, Austria, Brazil, Denmark, France, Germany, Hungary, Italy, Slovenia) | July 2015 – August 2015 | Pre-enrichment, selective culture (TSB with cefoxitin and aztreonam), MRSA ID chromogenic medium screening | 80 | 6 |
| 37 | Raji | 2016 | Saudi Arabia | Camel meat, beef, lamb/mutton | Retail supermarket chains, neighborhood butcher shops | March to December 2014 | Double broth enrichment technique, MRSA chromogenic medium, cefoxitin resistance, DNA microarray detection of mecA and specific SCCmec types | 71 | 12 |
|  | Raji | 2016 | Saudi Arabia | Chicken | Retail supermarket chains, neighborhood butcher shops | March to December 2014 | Double broth enrichment technique, MRSA chromogenic medium, cefoxitin resistance, DNA microarray detection of mecA and specific SCCmec types | 29 | 1 |
| 38 | Rahimi | 2016 | Iran | Chicken | Isfahan local market | July to December 2014 | Chromogenic medium screening, PCR for detecting mecA gene | 36 | 25 |
| 39 | Sallam | 2015 | Egypt | Chicken (whole chicken, chicken legs, gizzards, chicken liver) | Retail supermarkets and poultry stores | July-November 2012 | PCR for detection of mecA gene, confirmation of nuc and coa genes | 200 | 76 |
| 40 | Dhup | 2015 | England | Chicken | Pre-packaged meat in supermarkets and loose meat in butcher shops | March 2011 | Chromogenic medium screening, PCR for detecting mecA gene | 30 | 3 |
|  | Dhup | 2015 | England | Pork, beef | Pre-packaged meat in supermarkets and loose meat in butcher shops | March 2011 | Chromogenic medium screening, PCR for detecting mecA gene | 60 | 2 |
| 41 | Beninati | 2015 | Germany | Chicken, turkey | Retail supermarkets (imported from Italy) | NA | MRSA culture medium, Micronaut-s MRSA/IFSG GP 4 confirmed | 38 | 4 |
| 42 | Abdalrahman | 2015 | America | Chicken, turkey | Several grocery stores (conventional, organic) | January to June 2010 | PCR For detection of mecA gene, PFGE, spa typing and MLST molecular typing | 167 | 2 |
| 43 | Zarfel | 2014 | Austria | Chicken | Four different supermarket chains and a butcher | January to March 2012 | OXA agar screening for methicillin-resistant Staphylococci, PCR for detecting mecA gene | 50 | 0 |
| 44 | Vossenkuhl | 2014 | Germany | Turkey | Retail market | 2010 | OXA agar screening for methicillin-resistant Staphylococci, PCR for detecting mecA gene | 460 | 147 |
| 45 | Jackson | 2013 | America | Pork (chops, ground pork, pig ears, etc.), Beef (steaks, roasts, ground beef, etc.) | Retail meat (local grocery store) | 2009 (within 12 weeks) | Broth microdilution susceptibility testing, multiplex PCR for detecting mecA gene | 200 | 7 |
| 46 | Hu | 2013 | China | Cooked meat products (pork, beef, chicken, duck) | 4 hotels in Anhui | April to November 2011 | Antibiotic susceptibility testing by Kirby-Bauer method (KB method) | 40 | 0 |
| 47 | O'Brien | 2012 | America | Pork (pork chops, ground pork, ribs, sausages, pork tenderloin, pork loin, etc.) | 36 retail stores (supermarkets, specialty food markets, cooperative grocery stores) in different areas | September to October 2010 | Baird Parker agar culture, CHROMagar MRSA test, PBP2' latex agglutination test, PCR for detecting mecA gene | 395 | 26 |
| 48 | Molla | 2012 | America | Retail pork | Retail stores | NA | ORSA selective culture, mecA and nuc gene duplex PCR | 135 | 5 |
| 49 | Hiroi | 2012 | Japan | Chicken | Raw retail meat purchased from supermarkets or meat markets | 2004 to 2006 | PCR detection of mecA gene confirmation, E-test detection (oxacillin resistance) | 100 | 4 |
|  | Hiroi | 2012 | Japan | Pork, beef | Raw retail meat purchased from supermarkets or meat markets | 2004 to 2006 | PCR for mecA gene detection and confirmation, E-test method detection (oxacillin resistance) | 200 | 5 |
| 50 | Kelman | 2011 | America | Ground beef, ground pork | The three major supermarket chains in the Washington, D.C. area | March 2008 to August 2008 | Susceptibility testing (Sensititre broth microdilution and methicillin agar dilution), PCR for detection of mecA gene, PFGE typing | 498 | 1 |
|  | Kelman | 2011 | America | C rushed turkey | The three major supermarket chains in the Washington, D.C. area | March 2008 to August 2008 | Susceptibility testing (Sensititre broth microdilution and methicillin agar dilution), PCR for detection of mecA gene, PFGE typing | 196 | 0 |
| 51 | Hanson | 2011 | America | Pork, beef | 22 food stores in Iowa | February 2009 to April 2009 | MRSA latex agglutination test, PCR for detecting mecA gene | 84 | 2 |
|  | Hanson | 2011 | America | Chicken, turkey | 22 food stores in Iowa | February 2009 to April 2009 | MRSA latex agglutination test, PCR for detecting mecA gene | 81 | 0 |
| 52 | Feßler | 2011 | Germany | Fresh chicken, turkey | Retail store (Rhineland-Palatinate, Germany) | May 2009 to December 2009 | Chromogenic MRSA selective agar culture, PCR for detecting mecA gene | 46 | 17 |
|  | Feßler | 2011 | Germany | F resh chicken, turkey, chicken products, t urkey meat products | Retail store (Rhineland-Palatinate, Germany) | May 2009 to December 2009 | Chromogenic MRSA selective agar culture, PCR for detecting mecA gene | 40 | 15 |
| 53 | Bhargava | 2011 | America | Beef | 30 grocery stores in Detroit, Michigan | August 2009–January 2010 | Drug sensitivity test, PCRmecA gene detection | 156 | 2 |
|  | Bhargava | 2011 | America | Chicken, turkey | 30 grocery stores in Detroit, Michigan | August 2009–January 2010 | Drug sensitivity test, PCRmecA gene detection | 133 | 4 |
| 54 | Weese | 2010 | Canada | Pork chops, ground pork, pork shoulder | Retail stores in four provinces | August 2008 to November 2008 | PBP2a latex agglutination test and molecular typing | 402 | 31 |
| 55 | Huber | 2010 | Switzerland | Ground pork and ground beef | Foods of animal origin | March 2009 to September 2009 | MRSA chromogenic agar culture, PCR for detecting mecA gene | 160 | 0 |
| 56 | Pu | 2009 | America | Pork, beef | 30 retail supermarkets, covering 7 supermarket chain brands | February 2008 to March 2008 | Baird-Parker agar culture (with/without cefoxitin), PCR for detecting mecA gene | 120 | 6 |
| 57 | Loo | 2007 | Netherlands | Pork, beef | Supermarkets, butcher shops | February to May 2006 | Direct inoculation, enrichment culture (MRSA ID medium), PCR for detecting mecA gene | 79 | 2 |
| 58 | Savariraj | 2019 | India | Pork | 120 retail pork stores in four regions | NA | PCR for detecting mecA and mecC genes | 120 | 70 |
| 59 | Rahimi | 2019 | Iran | Beef | 27 grocery stores in Tehran | January to June 2016 | PCR using oxacillin and cefoxitin discs for detecting nucA and mecA genes | 62 | 29 |
|  | Rahimi | 2019 | Iran | Chicke, turkey | 27 grocery stores in Tehran | January to June 2016 | PCR using oxacillin and cefoxitin discs for detecting nucA and mecA genes | 69 | 20 |
| 60 | Velasco | 2018 | Chile | Pork | 3 supermarkets and 11 retail stores | NA | PCR for detecting nuc and mecA genes, PBP2' protein agglutination test | 70 | 0 |
| 61 | Bayomi | 2016 | Egypt | Chicken breast | Local retail markets and processing shops | NA | PCR for detecting mecA gene | 40 | 1 |
|  | Bayomi | 2016 | Egypt | Sliced luncheon meat, fried chicken nuggets | Local retail markets and processing shops | NA | PCR for detecting mecA gene | 40 | 6 |
| 62 | Teramoto | 2016 | America | Poultry (whole poultry carcasses) | 7 farmers markets, 3 organic retail supermarkets, 3 traditional retail supermarkets | February to September 2014 | PCR for detection (nuc gene), drug sensitivity test (agar dilution method) | 96 | 1 |
| 63 | Chairat | 2015 | Tunisia | Chicken | Markets, slaughterhouses, farms, supermarkets | October 2010–March 2011 | Disc diffusion method, PCR for detecting mecA gene | 84 | 2 |
|  | Chairat | 2015 | Tunisia | Lamb, veal, horsemeat, rabbit meat, pork | Markets, slaughterhouses, farms, supermarkets | October 2010–March 2011 | Disc diffusion method, PCR for detecting mecA gene | 80 | 0 |
| 64 | Benito | 2014 | Spain | Pork, beef, lamb | Supermarkets, small butcher shops | 2011 to 2012 | ORSA plate selective culture, PCR for detecting mecA gene | 54 | 1 |
|  | Benito | 2014 | Spain | Chicken, turkey | Supermarkets, small butcher shops | 2011 to 2012 | ORSA plate selective culture, PCR for detecting mecA gene | 46 | 0 |
| 65 | He | 2013 | China | Pork | Two supermarkets in Jinan | June 24, 2011 | Cefoxitin and oxacillin disk, PCR | 30 | 0 |
|  | He | 2013 | China | Chicken | Two supermarkets in Jinan | June 24, 2011 | Cefoxitin and oxacillin disk, PCR | 30 | 0 |
| 66 | Boost | 2013 | China | Pork, beef | Wet markets, supermarkets | 3 months | MRSA plate, PCR for detecting mecA gene | 735 | 31 |
|  | Boost | 2013 | China | Pork and beef balls | Wet markets, supermarkets | 3 months | MRSA plate, PCR for detecting mecA gene | 210 | 1 |
|  | Boost | 2013 | China | Chicken | Wet markets, supermarkets | 3 months | MRSA plate, PCR for detecting mecA gene | 455 | 95 |
| 67 | Guran | 2015 | Turkey | Beef, lamb | Retail stores | September 2013–March 2014 | Cefoxitin Kirby-Bauer method (CLSI method), PCR for detecting mecA gene | 250 | 40 |
| 68 | Petternel | 2014 | Austria | Mixed ground meat (pork and beef) | Supermarket and butcher shop in Graz | 2011 –September 2012 | Shimadzu MALDI-TOF MS AximaTM Assurance | 100 | 9 |
| 69 | Aklilu | 2016 | Malaysia | Raw chicken | Wet markets (three markets in Kota Bharu area) | NA | Selective culture medium (Brilliance MRSA2 agar), drug sensitivity test (CLSI standard), PCR for detecting mecA gene | 100 | 43 |
| 70 | Krumova-Valcheva | 2024 | Bulgaria | Raw pork | Retail network (import from Bulgaria and other countries) | August 2019 to December 2021 | Kirby-Bauer method (CLSI standard), minimum inhibitory concentration (MIC) determination, PCR for detecting mecA/mecC genes | 169 | 8 |
| 71 | Kanaan | 2019 | Iraq | Beef Imported and Local | Local butchers and supermarkets | March to June 2018 | Kirby-Bauer method, PBP2a latex agglutination test, SPOT Staphylect Plus kit | 75 | 9 |
| 72 | Koláčková​​ | 2014 | Czech Republic | Pork | 16 retail stores and supermarkets | 2012 to 2013 | MRSA 2 agar, PCR for detecting mecA gene | 197 | 2 |
| 73 | Zehra | 2019 | India | Chicken | Retail butcher shop | NA | PCR for detecting mecA gene | 147 | 4 |
|  | Zehra | 2019 | India | Pork, goat | Retail butcher shop | NA | PCR for detecting mecA gene | 261 | 0 |
| 74 | Weese | 2010 | Canada | Pork (pork chops, ground pork), ground beef | Retail stores (supermarket chains, independent markets/butchers) in four provinces | November 2008 to August 2009 | MRSA Chromogenic Agar | 428 | 33 |
|  | Weese | 2010 | Canada | Chicken (chicken drumstick, chicken wing, chicken thigh) | Retail stores (supermarket chains, independent markets/butchers) in four provinces | November 2008 to August 2009 | MRSA Chromogenic Agar | 250 | 3 |
| 75 | Wang | 2013 | China | Chicken​ | Retail market | March to December 2010 | PCR for detection of mecA gene confirmation | 1152 | 20 |
| 76 | Tang | 2017 | Denmark | Chicken (Danish), fire chicken (non-Danish) | Supermarkets (pre-packaged fresh meat from industrial slaughterhouses) | November 2014 to September 2015 | Enrichment detection method (MRSA2 agar), confirmation method: MALDI-TOF MS identification, PCR detection of mecA and spa genes | 125 | 16 |
|  | Tang | 2017 | Denmark | Pork (danish) | Danish supermarkets (pre-packaged fresh meat from industrial slaughterhouses) | November 2014 to September 2015 | Enrichment detection method (MRSA2 agar), confirmation method: MALDI-TOF MS identification, PCR for detecting mecA and spa genes | 20 | 3 |
| 77 | Sun | 2019 | China | Retail pork) | Pork wholesale market | April 2016 | CHROMagar MRSA isolation, MALDI-TOF MS identification, PCR for confirming the mecA gene | 14 | 2 |
| 78 | Pauly | 2019 | Germany | Chicken (skin-on chicken wings, drumsticks, whole chicken) | Supermarket retail in Berlin and surrounding areas | January to March 2018 | Confirmation method: MALDI-TOF MS identification, PCR for detection of mecA and nuc genes | 215 | 36 |
| 79 | Narvaez-Bravo | 2016 | Canada | Pork | Commercial pork processing plant | September 2010 to August 2011 | Selective MRSA chromogenic agar plates, MRSA latex agglutination test, PCR for detecting mecA gene | 660 | 8 |
| 80 | Mama | 2020 | Spain | Pork (ground meat, tenderloin, ears/nose) | Retail butcher shop | March to October 2018 | MALDI-TOF mass spectrometry identification, cefoxitin resistance screening, PCR for detecting mecA gene | 101 | 22 |
| 81 | Lozano | 2009 | Spain | Pork, veal, lamb, rabbit meat | Retail market in La Rioja | November 2007–March 2009 | ORSAB plate (containing cefoxitin) enrichment culture, PCR for detecting mecA and nuc genes | 128 | 3 |
|  | Lozano | 2009 | Spain | Chicken, turkey, | Retail market in La Rioja | November 2007 to March 2009 | ORSAB plate (containing cefoxitin) enrichment culture, PCR for detecting mecA and nuc genes | 158 | 1 |
| 82 | Liu | 2019 | China | Chilled pork | Chinese e-commerce platforms (Alibaba, Taobao, JD.com, etc.) | May to September 2017 | MRSA culture medium isolation, PCR for detection of mecA gene, confirmed by VITEK 2 Compact system | 135 | 5 |
| 83 | Qiuchun Li | 2019 | China | Chicken | Supermarkets, farmers' markets, wholesale markets, slaughterhouses, vegetable/food markets | July 2016 to November 2016 | Cefoxitin Kirby-Bauer method, PCR for detecting mecA gene | 507 | 8 |
| 84 | Lili | 2016 | China | Raw pork | Supermarkets and butcher shops in Guangzhou | September 2013 to November 2013 | PCR for detection of mecA gene, Kirby-Bauer disk diffusion method | 240 | 10 |
|  | Lili | 2016 | China | Cooked pork products | Supermarkets and butcher shops in Guangzhou | September 2013 to November 2013 | PCR For detection of mecA gene, Kirby-Bauer method | 240 | 0 |
| 85 | Heng Li | 2021 | China | Pork | Supermarkets in Beijing | October 2019 to November 2019 | Phenotypic screening (cefoxitin resistance), PCR for detecting mecA gene | 8 | 1 |
| 86 | Ge | 2016 | America | Beef, pork | 345 stores in different regions | 2010 to 2011 | Selective culture, phenotypic identification, PCR for genetic testing | 1760 | 32 |
|  | Ge | 2016 | America | Chicken, turkey | 345 stores in different regions | 2010 to 2011 | Selective culture, phenotypic identification, PCR for genetic testing | 1760 | 34 |
| 87 | Fox | 2017 | England | Pork | Different butcher shops and supermarkets | March to July 2015 | MRSA 2 agar, PCR for detecting mecA and nuc genes | 63 | 3 |
|  | Fox | 2017 | England | Chicken, turkey | Different butcher shops and supermarkets | March to July 2015 | MRSA 2 agar, PCR for detecting mecA and nuc genes | 61 | 6 |
| 88 | Boer | 2009 | Netherlands | Beef, pork, veal, lamb/mutton | Retail trade | June 2007 to May 2008 | MRSA agar after enrichment, latex agglutination test, PCR for detecting mecA gene | 1285 | 134 |
|  | Boer | 2009 | Netherlands | Chicken, turkey, poultry | Retail trade | June 2007 to May 2008 | MRSA agar after enrichment, latex agglutination test, PCR for detecting mecA gene | 754 | 128 |
| 89 | Costa | 2015 | Brazil | Raw meat: beef, pork | Public hospital kitchen | July 2011 to May 2012 | Selective medium ( MRSA-ID) isolation, Detection of PBP2a by latex agglutination test | 54 | 16 |
|  | Costa | 2015 | Brazil | Raw meat: chicken | Public hospital kitchen | July 2011 to May 2012 | selective culture medium (MRSA-ID), detection of PBP2a by latex agglutination test | 30 | 7 |
| 90 | Cho | 2014 | Korea | Beef, pork | 51 randomly selected wholesale markets in different regions | February to October 2011 | Kirby-Bauer method, PCR for detecting mecA gene | 114 | 1 |
|  | Cho | 2014 | Korea | Chicken | 51 randomly selected wholesale markets in different regions | February to October 2011 | Kirby-Bauer method, PCR for detecting mecA gene | 41 | 2 |
| 91 | Buyukcangaz | 2013 | America | Pork, beef | Four supermarket chains | May 2010 to April 2011 | PCR for confirming the mecA gene | 108 | 5 |
|  | Buyukcangaz | 2013 | America | Chicken | Four supermarket chains | May 2010 to April 2011 | PCR for confirming the mecA gene | 37 | 0 |
|  | Buyukcangaz | 2013 | America | Deli meats (ham, turkey, chicken) | Four supermarket chains | May 2010 to April 2011 | PCR for confirming the mecA gene | 46 | 0 |
| 92 | Abolghait | 2020 | Egypt | Chicken (breast, leg, liver, gizzard) | 18 different retail markets | February to November 2018 | Cefoxitin Kirby-Bauer for screening, PCR for confirming mecA and mecC genes | 144 | 8 |
| 93 | Ruban | 2018 | India | Buffalo meat | Different retail stores | NA | PCR for detecting femA and mecA genes | 40 | 13 |
| 94 | Saadati | 2021 | Iran | Chicken, turkey, quail, ostrich | Malls in Tehran Province | May to August 2018 | Cefoxitin and oxacillin susceptibility testing, PCR for detecting mecA gene | 240 | 12 |
| 95 | Gokmen | 2024 | Turkey | Beef | Balıkesir market | NA | PCR for detecting nuc and mecA genes | 100 | 6 |
|  | Gokmen | 2024 | Turkey | Chicken, turkey | Balıkesir market | NA | PCR for detecting nuc and mecA genes | 150 | 4 |
| 96 | Lim | 2010 | Korea | Beef, pork | 25 retail markets | 2003 to 2008 | Kirby-Bauer method and E-test method were used to determine the minimum inhibitory concentration (MIC), PCR for detecting mecA gene | 99 | 9 |
|  | Lim | 2010 | Korea | Chicken | 25 retail markets | 2003 to 2008 | Kirby-Bauer method and E-test method were used to determine the minimum inhibitory concentration (MIC), PCR for detecting mecA gene | 11 | 0 |
| 97 | Gelbí č ová | 2022 | Czech Republic | Beef tartare, shelf-stable fermented meat products (pork and beef), dried meat products (pork, beef, poultry and venison) | Retail stores | NA | MRSA agar, PCR for detecting SA442 fragment and mecA gene | 84 | 4 |
|  | Gelbí č ová | 2022 | Czech Republic | Heat-treated meat products (pork and poultry), shelf-stable heat-treated meat products (pork and beef) | Retail stores | NA | MRSA agar, PCR for detecting SA442 fragment and mecA gene | 97 | 0 |
| 98 | Chan | 2008 | America | Beef, pork | 10 butcher shops and supermarkets in the Providence, Rhode Island Area | December 15, 2007 | Oxacillin agar plates, uncertain delivery confirmation | 24 | 0 |
|  | Chan | 2008 | America | Chicken | 10 butcher shops and Supermarkets in the Providence, Rhode Island Area | December 15, 2007 | Oxacillin agar plates, uncertain delivery confirmation | 12 | 0 |

**Table S3** Quality assessments using the JBI checklist.

| NO | Author | Year | 1.Was the sample frame appropriate to address the target opulation? | 2.Were study participants sampled in an appropriate way? | 3.Was the sample size adequate? | 4.Were the study subjects and the setting described in detail? | 5.Was the data analysis conducted with sufficient coverage of the identified sample? | 6.Were valid methods used for the identification of the condition? | 7.Was the condition measured in a standard, reliable way for all participants? | 8.Was there appropriate statistical analysis? | 9.Was the response rate adequate, and if not, was the low response rate managed appropriately? | score |
| --- | --- | --- | --- | --- | --- | --- | --- | --- | --- | --- | --- | --- |
| 1 | Sheet | 2024 | Y | Y | Y | Y | Y | Y | Y | Y | NA | 8 |
| 2 | Kizanlik | 2024 | Y | Y | Y | Y | Y | Y | Y | Y | NA | 8 |
| 3 | Gonzalez-Fandos | 2024 | Y | Y | N | Y | Y | Y | Y | Y | NA | 7 |
| 4 | Eguizába | 2024 | Y | Y | Y | Y | Y | Y | Y | Y | NA | 8 |
| 5 | Guedes | 2024 | Y | Y | Y | Y | Y | Y | Y | Y | NA | 8 |
| 6 | Sheet | 2023 | Y | Y | Y | Y | Y | Y | Y | Y | NA | 8 |
| 7 | Naeim | 2023 | Y | Y | N | Y | Y | Y | Y | Y | NA | 7 |
| 8 | Morshdy | 2023 | Y | Y | Y | Y | Y | Y | Y | Y | NA | 8 |
| 9 | Martinez-Laorden | 2023 | Y | Y | Y | Y | Y | Y | Y | Y | NA | 8 |
| 10 | Igbinosa | 2023 | Y | Y | Y | Y | Y | Y | Y | Y | NA | 8 |
| 11 | Dorigochoo | 2023 | Y | Y | Y | Y | Y | Y | Y | Y | NA | 8 |
| 12 | Zhu | 2022 | Y | Y | Y | Y | Y | Y | Y | Y | NA | 8 |
| 13 | Thwala | 2022 | Y | Y | Y | Y | Y | Y | Y | Y | NA | 8 |
| 14 | Osada | 2022 | Y | Y | Y | Y | Y | Y | Y | Y | NA | 8 |
| 15 | Komodromos | 2022 | Y | Y | Y | Y | Y | Y | Y | Y | NA | 8 |
| 16 | Guran | 2022 | Y | Y | Y | Y | Y | Y | Y | Y | NA | 8 |
| 17 | Telli | 2021 | Y | Y | N | Y | Y | Y | Y | Y | NA | 7 |
| 18 | Tegegne | 2021 | Y | Y | Y | Y | Y | Y | Y | Y | NA | 8 |
| 19 | Tanomsridachchai | 2021 | Y | Y | Y | Y | Y | Y | Y | Y | NA | 8 |
| 20 | Parvin | 2021 | Y | Y | Y | Y | Y | Y | Y | Y | NA | 8 |
| 21 | Martínez-Vázquez | 2021 | Y | Y | Y | Y | Y | Y | Y | Y | NA | 8 |
| 22 | Sankomkai | 2020 | Y | Y | Y | Y | Y | Y | Y | Y | NA | 8 |
| 23 | Sadiq | 2020 | Y | Y | Y | Y | Y | Y | Y | Y | NA | 8 |
| 24 | Ogundipe | 2020 | Y | Y | Y | Y | Y | Y | Y | Y | NA | 8 |
| 25 | Kim | 2020 | Y | Y | Y | Y | Y | Y | Y | Y | NA | 8 |
| 26 | Bouchami | 2020 | Y | Y | N | Y | Y | Y | Y | Y | NA | 7 |
| 27 | Bernier-Lachance | 2020 | Y | Y | Y | Y | Y | Y | Y | Y | NA | 8 |
| 28 | Basanisi | 2020 | Y | Y | Y | Y | Y | Y | Y | Y | NA | 8 |
| 29 | Sivakumar | 2019 | Y | Y | Y | Y | Y | Y | Y | Y | NA | 8 |
| 30 | Naas | 2019 | Y | Y | Y | Y | Y | Y | Y | Y | NA | 8 |
| 31 | El-Ghareeb | 2019 | Y | Y | Y | Y | Y | Y | Y | Y | NA | 8 |
| 32 | Kim | 2018 | Y | Y | Y | Y | Y | Y | Y | Y | NA | 8 |
| 33 | Thapaliya | 2017 | Y | Y | Y | Y | Y | Y | Y | Y | NA | 8 |
| 34 | Osman | 2017 | Y | Y | Y | Y | Y | Y | Y | Y | NA | 8 |
| 35 | Arslan | 2017 | Y | Y | Y | Y | Y | Y | Y | Y | NA | 8 |
| 36 | Zogg | 2016 | Y | Y | Y | Y | Y | Y | Y | Y | NA | 8 |
| 37 | Raji | 2016 | Y | Y | Y | Y | Y | Y | Y | Y | NA | 8 |
| 38 | Rahimi | 2016 | Y | Y | Y | Y | Y | Y | Y | Y | NA | 8 |
| 39 | Sallam | 2015 | Y | Y | Y | Y | Y | Y | Y | Y | NA | 8 |
| 40 | Dhup | 2015 | Y | Y | Y | Y | Y | Y | Y | Y | NA | 8 |
| 41 | Beninati | 2015 | Y | Y | Y | Y | Y | Y | Y | Y | NA | 8 |
| 42 | Abdalrahman | 2015 | Y | Y | Y | Y | Y | Y | Y | Y | NA | 8 |
| 43 | Zarfel | 2014 | Y | Y | Y | Y | Y | Y | Y | Y | NA | 8 |
| 44 | Vossenkuhl | 2014 | Y | Y | Y | Y | Y | Y | Y | Y | NA | 8 |
| 45 | Jackson | 2013 | Y | Y | Y | Y | Y | Y | Y | Y | NA | 8 |
| 46 | Hu | 2013 | Y | Y | Y | Y | Y | Y | Y | Y | NA | 8 |
| 47 | O'Brien | 2012 | Y | Y | Y | Y | Y | Y | Y | Y | NA | 8 |
| 48 | Molla | 2012 | Y | Y | Y | Y | Y | Y | Y | Y | NA | 8 |
| 49 | Hiroi | 2012 | Y | Y | Y | Y | Y | Y | Y | Y | NA | 8 |
| 50 | Kelman | 2011 | Y | Y | Y | Y | Y | Y | Y | Y | NA | 8 |
| 51 | Hanson | 2011 | Y | Y | Y | Y | Y | Y | Y | Y | NA | 8 |
| 52 | Feßler | 2011 | Y | Y | Y | Y | Y | Y | Y | Y | NA | 8 |
| 53 | Bhargava | 2011 | Y | Y | Y | Y | Y | Y | Y | Y | NA | 8 |
| 54 | Weese | 2010 | Y | Y | Y | Y | Y | Y | Y | Y | NA | 8 |
| 55 | Huber | 2010 | Y | Y | Y | Y | Y | Y | Y | Y | NA | 8 |
| 56 | Pu | 2009 | Y | Y | Y | Y | Y | Y | Y | Y | NA | 8 |
| 57 | Loo | 2007 | Y | Y | Y | Y | Y | Y | Y | Y | NA | 8 |
| 58 | Savariraj | 2019 | Y | Y | Y | Y | Y | Y | Y | Y | NA | 8 |
| 59 | Rahimi | 2019 | Y | Y | Y | Y | Y | Y | Y | Y | NA | 8 |
| 60 | Velasco | 2018 | Y | Y | Y | Y | Y | Y | Y | Y | NA | 8 |
| 61 | Bayomi | 2016 | Y | Y | Y | Y | Y | Y | Y | Y | NA | 8 |
| 62 | Teramoto | 2016 | Y | Y | Y | Y | Y | Y | Y | Y | NA | 8 |
| 63 | Chairat | 2015 | Y | Y | Y | Y | Y | Y | Y | Y | NA | 8 |
| 64 | Benito | 2014 | Y | Y | Y | Y | Y | Y | Y | Y | NA | 8 |
| 65 | He | 2013 | Y | Y | Y | Y | Y | Y | Y | Y | NA | 8 |
| 66 | Boost | 2013 | Y | Y | Y | Y | Y | Y | Y | Y | NA | 8 |
| 67 | Guran | 2015 | Y | Y | Y | Y | Y | Y | Y | Y | NA | 8 |
| 68 | Petternel | 2014 | Y | Y | Y | Y | Y | Y | Y | Y | NA | 8 |
| 69 | Aklilu | 2016 | Y | Y | Y | Y | Y | Y | Y | Y | NA | 8 |
| 70 | Krumova-Valcheva | 2024 | Y | Y | Y | Y | Y | Y | Y | Y | NA | 8 |
| 71 | Kanaan | 2019 | Y | Y | Y | Y | Y | Y | Y | Y | NA | 8 |
| 72 | Koláčková | 2014 | Y | Y | Y | Y | Y | Y | Y | Y | NA | 8 |
| 73 | Zehra | 2019 | Y | Y | Y | Y | Y | Y | Y | Y | NA | 8 |
| 74 | Weese | 2010 | Y | Y | Y | Y | Y | Y | Y | Y | NA | 8 |
| 75 | Wang | 2013 | Y | Y | Y | Y | Y | Y | Y | Y | NA | 8 |
| 76 | Tang | 2017 | Y | Y | Y | Y | Y | Y | Y | Y | NA | 8 |
| 77 | Sun | 2019 | Y | Y | N | Y | Y | Y | Y | Y | NA | 7 |
| 78 | Pauly | 2019 | Y | Y | Y | Y | Y | Y | Y | Y | NA | 8 |
| 79 | Narvaez-Bravo | 2016 | Y | Y | Y | Y | Y | Y | Y | Y | NA | 8 |
| 80 | Mama | 2020 | Y | Y | Y | Y | Y | Y | Y | Y | NA | 8 |
| 81 | Lozano | 2009 | Y | Y | Y | Y | Y | Y | Y | Y | NA | 8 |
| 82 | Liu | 2019 | Y | Y | Y | Y | Y | Y | Y | Y | NA | 8 |
| 83 | Qiuchun Li | 2019 | Y | Y | Y | Y | Y | Y | Y | Y | NA | 8 |
| 84 | Lili Li | 2016 | Y | Y | Y | Y | Y | Y | Y | Y | NA | 8 |
| 85 | Heng Li | 2021 | Y | Y | N | Y | Y | Y | Y | Y | NA | 7 |
| 86 | Ge | 2016 | Y | Y | Y | Y | Y | Y | Y | Y | NA | 8 |
| 87 | Fox | 2017 | Y | Y | Y | Y | Y | Y | Y | Y | NA | 8 |
| 88 | Boer | 2009 | Y | Y | Y | Y | Y | Y | Y | Y | NA | 8 |
| 89 | Costa | 2015 | Y | Y | Y | Y | Y | Y | Y | Y | NA | 8 |
| 90 | Cho | 2014 | Y | Y | Y | Y | Y | Y | Y | Y | NA | 8 |
| 91 | Buyukcangaz | 2013 | Y | Y | Y | Y | Y | Y | Y | Y | NA | 8 |
| 92 | Abolghait | 2020 | Y | Y | Y | Y | Y | Y | Y | Y | NA | 8 |
| 93 | Ruban | 2018 | Y | Y | Y | Y | Y | Y | Y | Y | NA | 8 |
| 94 | Saadati | 2021 | Y | Y | Y | Y | Y | Y | Y | Y | NA | 8 |
| 95 | Gökmen | 2024 | Y | Y | Y | Y | Y | Y | Y | Y | NA | 8 |
| 96 | Lim | 2010 | Y | Y | N | Y | Y | Y | Y | Y | NA | 7 |
| 97 | Gelbíčová | 2022 | Y | Y | Y | Y | Y | Y | Y | Y | NA | 8 |
| 98 | Chan | 2008 | Y | Y | N | Y | Y | Y | Y | Y | NA | 7 |


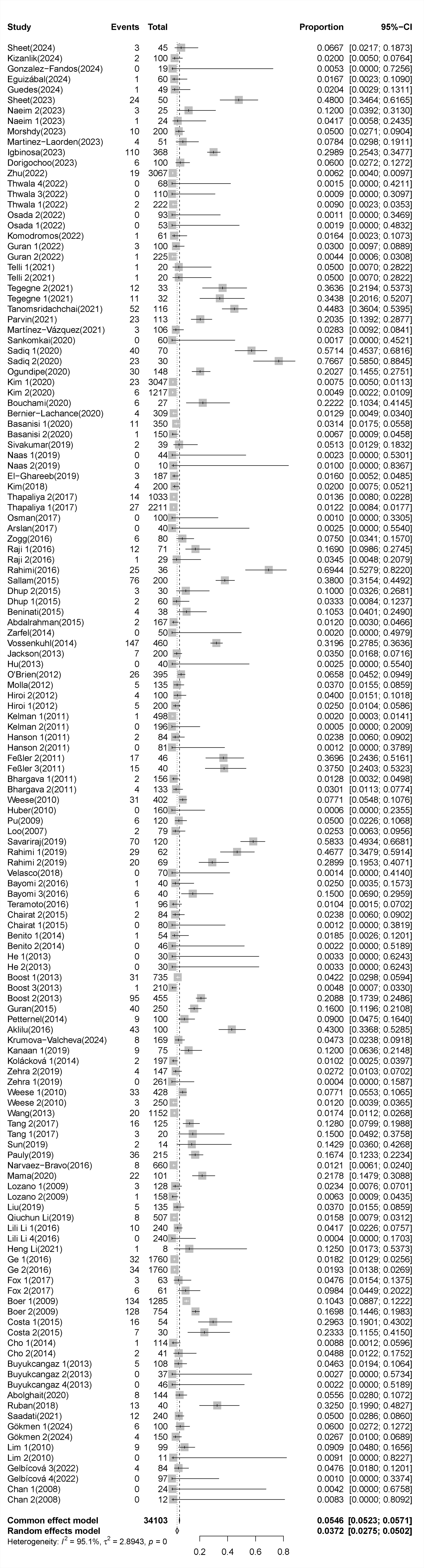


**Figure S1** Forest Plot.


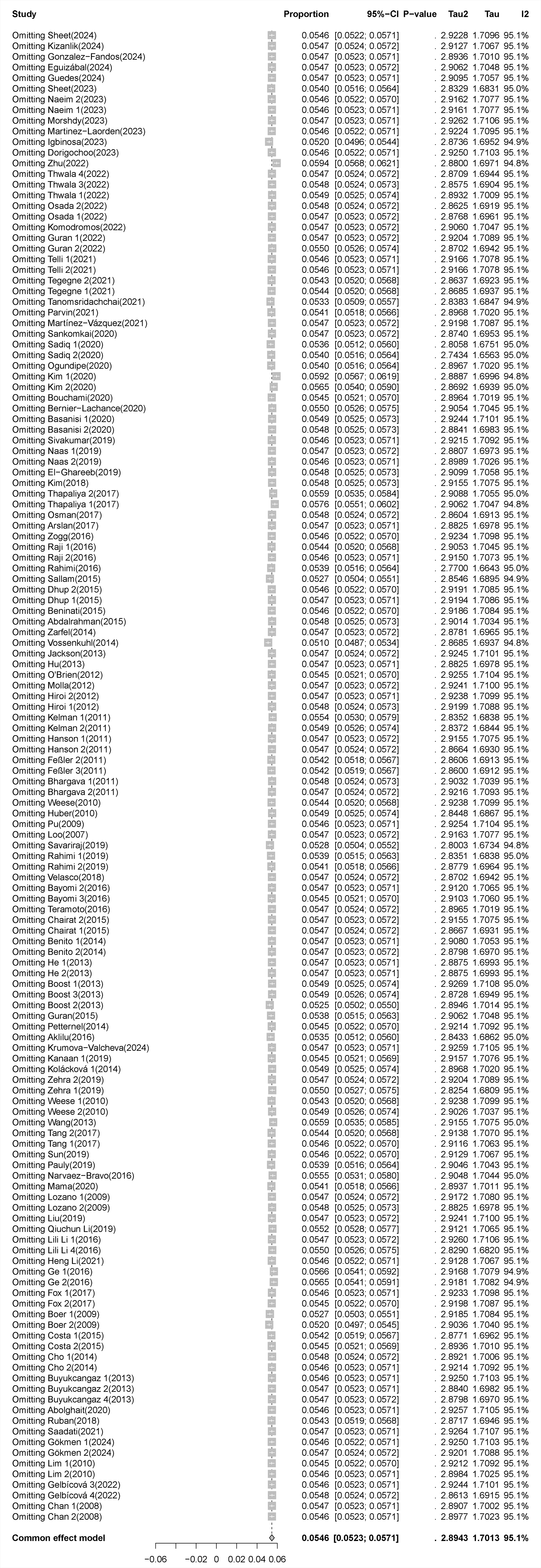


**Figure S2** Sensitivity Analysis Plot.


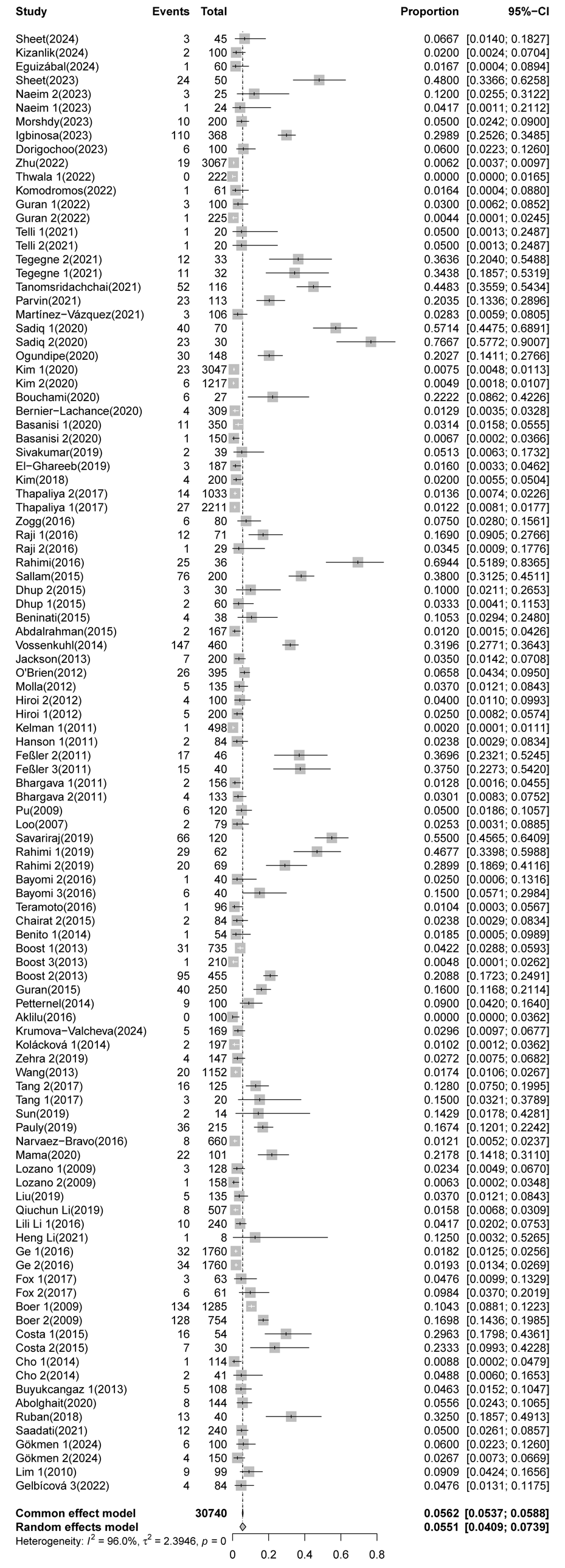


**Figure S3** Forest Plot (mec A).


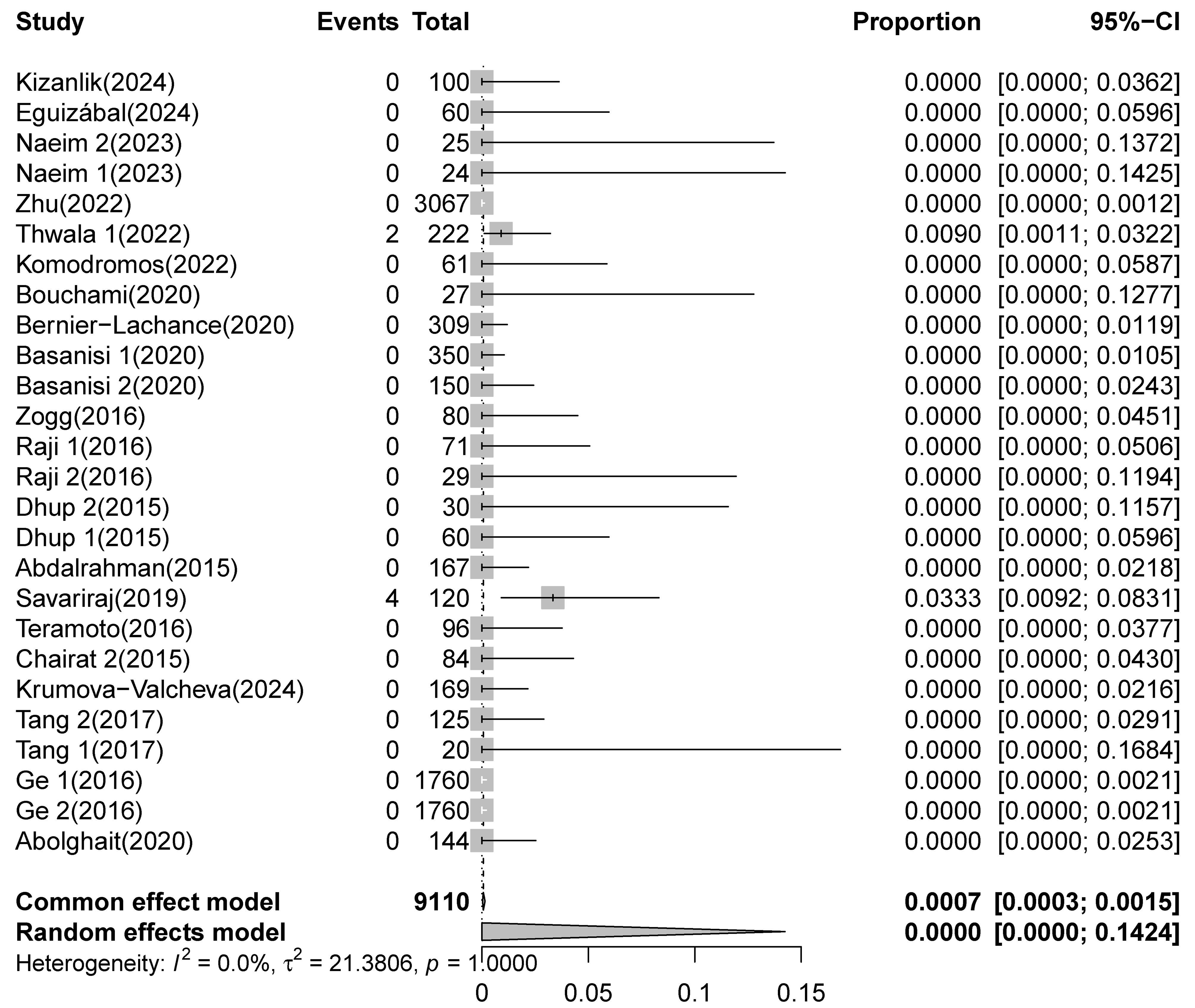


**Figure S4** Forest Plot (mec C).


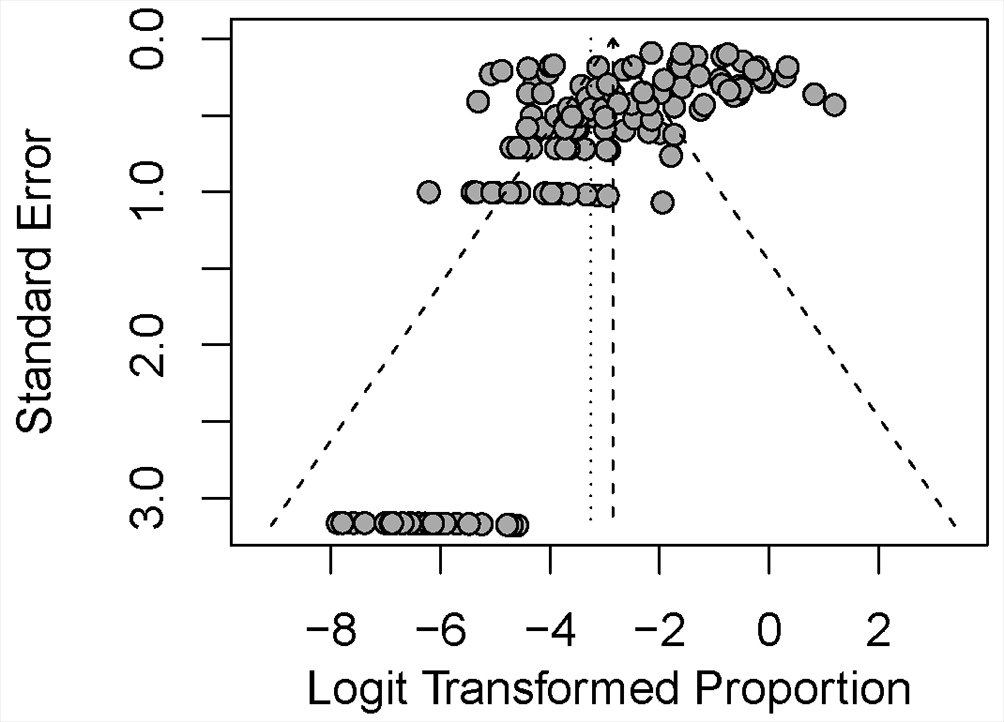


**Figure S5** Funnel Plot.


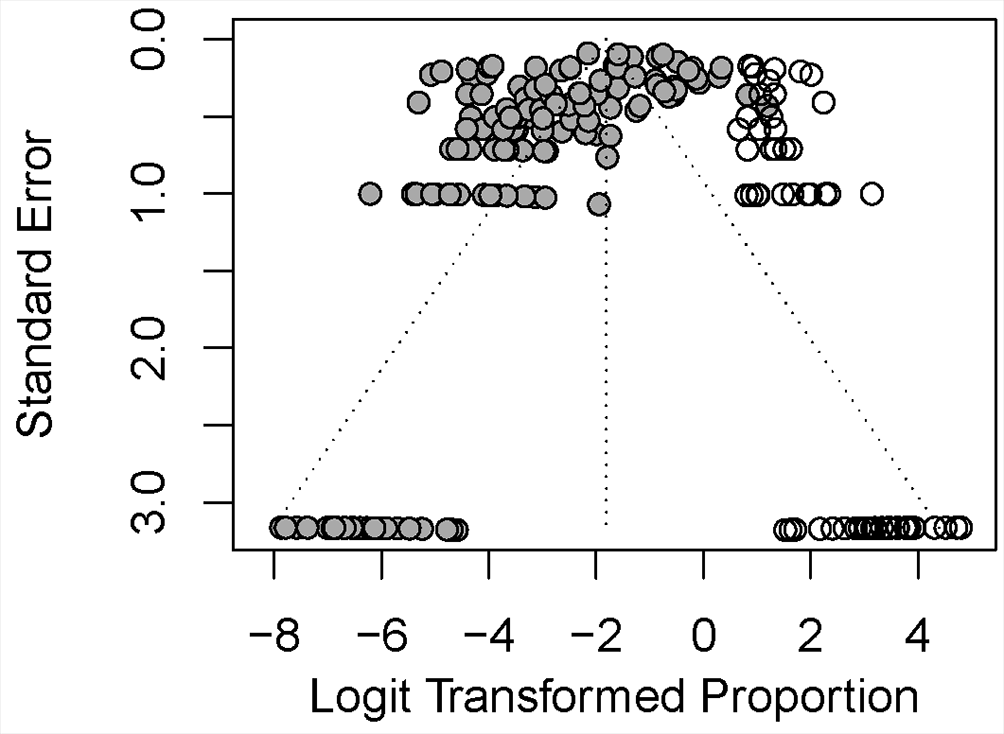


**Figure S6** Trim-and-Fill Funnel Plot.(/ Adjusted funnel plot.)

**File S1** The 98 included references

1. Abbasi K, Tajbakhsh E, Momtaz H. Antimicrobial resistance, virulence genes, and biofilm formation in Staphylococcus aureus strains isolated from meat and meat products. *Journal of Food Safety*. (2021) 41(6):e12933. doi: 10.1111/jfs.12933

2. Kizanlik PK, Goksoy EO. The prevalence, enterotoxigenic properties and antimicrobial susceptibility of Staphylococcus aureus isolated from various foods of animal origin. *Veterinary Archives*. (2024) 94(1):43-54. doi: 10.24099/vet.arhiv.1987

3. Gonzalez-Fandos E, da Silva Guedes J. Microbiological Quality and Antibiotic Resistance of Relevant Bacteria from Horsemeat. *Microorganisms*. (2024) 12(9):1775. doi: 10.3390/microorganisms12091775

4. Eguizábal P, Fernández-Fernández R, Campaña-Burguet A, González-Azcona C, Marañón-Clemente I, Tenorio C, et al. High prevalence of avian adapted CC5 Staphylococcus aureus isolates in poultry meat in Spain: food chain as vehicle of MRSA and MSSA CC398-t1451. *International Journal of Food Science & Technology*. (2024) 59(12):9180-8. doi: 10.1111/ijfs.17521

5. da Silva Guedes J, Velilla-Rodriguez D, González-Fandos E. Microbiological Quality and Safety of Fresh Rabbit Meat with Special Reference to Methicillin-Resistant S. aureus (MRSA) and ESBL-Producing E. coli. *Antibiotics*. (2024) 13(3):256. doi: 10.3390/antibiotics13030256

6. Sheet O, Al-Mahmood OA, Othman SM, Alsanjary RA. Detection of positive mecA Staphylococcus aureus isolated from meat and butchers' shops by using PCR technique in Mosul city. *Iraqi Journal of Veterinary Sciences*. (2023) 37(4):865-70. doi:

7. Elsayed Naeim D, Elsayed Eldesoukey I, Ahmed Moawad A, Mohammed Ahmed A. Molecular detection of methicillin-resistant Staphylococcus aureus isolated from different foodstuffs in Egypt. *Vet Res Forum*. (2023) 14(5):243-8. doi: 10.30466/vrf.2022.551346.3434

8. Morshdy AEMA, Tharwat AE, Merwad AMA, Abdallah NAM, Saber T. PREVALENCE, PHENOTYPIC-GENOTYPIC RESISTANCE AND BIOFILM FORMATION OF Staphylococcus aureus IN CHICKEN MEAT WITH REFERENCE TO ITS PUBLIC HEALTH HAZARD. *Slovenian Veterinary Research*. (2023) 60(25-Suppl):413–24. doi: 10.26873/SVR-1646-2022

9. Martínez-Laorden A, Arraiz-Fernández C, González-Fandos E. Microbiological Quality and Safety of Fresh Turkey Meat at Retail Level, Including the Presence of ESBL-Producing Enterobacteriaceae and Methicillin-Resistant S. aureus. *Foods*. (2023) 12(6). doi: 10.3390/foods12061274

10. Igbinosa EO, Beshiru A, Igbinosa IH, Ogofure AG, Ekundayo TC, Okoh AI. Prevalence, multiple antibiotic resistance and virulence profile of methicillin-resistant Staphylococcus aureus (MRSA) in retail poultry meat from Edo, Nigeria. *Front Cell Infect Microbiol*. (2023) 13:1122059. doi: 10.3389/fcimb.2023.1122059

11. Dorjgochoo A, Batbayar A, Tsend-Ayush A, Erdenebayar O, Byambadorj B, Jav S, et al. Detection of virulence genes of Staphylococcus aureus isolated from raw beef for retail sale in the markets of Ulaanbaatar city, Mongolia. *BMC Microbiol*. (2023) 23(1):372. doi: 10.1186/s12866-023-03122-2

12. Zhu Z, Liu X, Chen X, Zou G, Huang Q, Meng X, et al. Prevalence and Virulence Determinants of Staphylococcus aureus in Wholesale and Retail Pork in Wuhan, Central China. *Foods*. (2022) 11(24). doi: 10.3390/foods11244114

13. Thwala T, Madoroba E, Maliehe TS, Magwedere K, Basson AK, Butaye P. Antimicrobial Resistance, Enterotoxin and mec Gene Profiles of Staphylococcus aureus Associated with Beef-Based Protein Sources from KwaZulu-Natal Province, South Africa. *Microorganisms*. (2022) 10(6). doi: 10.3390/microorganisms10061211

14. Osada M, Aung MS, Urushibara N, Kawaguchiya M, Ohashi N, Hirose M, et al. Prevalence and Antimicrobial Resistance of Staphylococcus aureus and Coagulase-Negative Staphylococcus/Mammaliicoccus from Retail Ground Meat: Identification of Broad Genetic Diversity in Fosfomycin Resistance Gene fosB. *Pathogens*. (2022) 11(4). doi: 10.3390/pathogens11040469

15. Komodromos D, Kotzamanidis C, Giantzi V, Pappa S, Papa A, Zdragas A, et al. Prevalence, Infectious Characteristics and Genetic Diversity of Staphylococcus aureus and Methicillin-Resistant Staphylococcus aureus (MRSA) in Two Raw-Meat Processing Establishments in Northern Greece. *Pathogens*. (2022) 11(11). doi: 10.3390/pathogens11111370

16. Guran HS, Bayrak ARB, Alali WQ, Yesiloglu C. Prevalence and antimicrobial susceptibility pattern of methicillin-resistant isolates from retail raw meats in Turkey. *International Food Research Journal*. (2022) 29(5):1089 - 100. doi: 10.47836/ifrj.29.5.11

17. Telli N, Telli AE, Biçer Y, Turkal G, Uçar G. Isolation and antimicrobial resistance of vancomycin resistant Enterococcus spp. (VRE) and methicillin-resistant S. aureus (MRSA) on beef and chicken meat, and workers hands from slaughterhouses and retail shops in Turkey. *Journal of the Hellenic Veterinary Medical Society*. (2021) 72(4):3345-54. doi: 10.12681/jhvms.29373

18. Tegegne HA, Koláčková I, Florianová M, Gelbíčová T, Madec JY, Haenni M, et al. Detection and molecular characterisation of methicillin-resistant Staphylococcus aureus isolated from raw meat in the retail market. *J Glob Antimicrob Resist*. (2021) 26:233-8. doi: 10.1016/j.jgar.2021.06.012

19. Tanomsridachchai W, Changkaew K, Changkwanyeun R, Prapasawat W, Intarapuk A, Fukushima Y, et al. Antimicrobial Resistance and Molecular Characterization of Methicillin-Resistant Staphylococcus aureus Isolated from Slaughtered Pigs and Pork in the Central Region of Thailand. *Antibiotics (Basel)*. (2021) 10(2). doi: 10.3390/antibiotics10020206

20. Parvin MS, Ali MY, Talukder S, Nahar A, Chowdhury EH, Rahman MT, et al. Prevalence and Multidrug Resistance Pattern of Methicillin Resistant S. aureus Isolated from Frozen Chicken Meat in Bangladesh. *Microorganisms*. (2021) 9(3). doi: 10.3390/microorganisms9030636

21. Martínez-Vázquez AV, Guardiola-Avila IB, Flores-Magallón R, Rivera G, Bocanegra-García V. Detection of multi-drug resistance and methicillin-resistant Staphylococcus aureus (MRSA) isolates from retail meat in Tamaulipas, Mexico. *Annals of Microbiology*. (2021) 71(1):16. doi: 10.1186/s13213-021-01627-7

22. Sankomkai W, Boonyanugomol W, Kraisriwattana K, Nutchanon J, Boonsam K, Kaewbutra S, et al. Characterisation of Classical Enterotoxins, Virulence Activity, and Antibiotic Susceptibility of Staphylococcus Aureus Isolated from Thai Fermented Pork Sausages, Clinical Samples, and Healthy Carriers in Northeastern Thailand. *J Vet Res*. (2020) 64(2):289-97. doi: 10.2478/jvetres-2020-0036

23. Sadiq A, Samad M, Saddam, Basharat N, Ali S, Roohullah, et al. Methicillin-Resistant Staphylococcus aureus (MRSA) in Slaughter Houses and Meat Shops in Capital Territory of Pakistan During 2018-2019. *Front Microbiol*. (2020) 11:577707. doi: 10.3389/fmicb.2020.577707

24. Ogundipe FO, Ojo OE, Feßler AT, Hanke D, Awoyomi OJ, Ojo DA, et al. Antimicrobial Resistance and Virulence of Methicillin-Resistant Staphylococcus aureus from Human, Chicken and Environmental Samples within Live Bird Markets in Three Nigerian Cities. *Antibiotics (Basel)*. (2020) 9(9). doi: 10.3390/antibiotics9090588

25. Kim YH, Kim HS, Kim S, Kim M, Kwak HS. Prevalence and Characteristics of Antimicrobial-Resistant Staphylococcus aureus and Methicillin-Resistant Staphylococcus aureus from Retail Meat in Korea. *Food Sci Anim Resour*. (2020) 40(5):758-71. doi: 10.5851/kosfa.2020.e50

26. Bouchami O, Fraqueza MJ, Faria NA, Alves V, Lawal OU, de Lencastre H, et al. Evidence for the Dissemination to Humans of Methicillin-Resistant Staphylococcus aureus ST398 through the Pork Production Chain: A Study in a Portuguese Slaughterhouse. *Microorganisms*. (2020) 8(12). doi: 10.3390/microorganisms8121892

27. Bernier-Lachance J, Arsenault J, Usongo V, Parent É, Labrie J, Jacques M, et al. Prevalence and characteristics of Livestock-Associated Methicillin-Resistant Staphylococcus aureus (LA-MRSA) isolated from chicken meat in the province of Quebec, Canada. *PLoS One*. (2020) 15(1):e0227183. doi: 10.1371/journal.pone.0227183

28. Basanisi MG, La Bella G, Nobili G, Tola S, Cafiero MA, La Salandra G. PREVALENCE AND CHARACTERIZATION OF METHICILLIN-RESISTANT STAPHYLOCOCCUS AUREUS (MRSA) ISOLATES FROM RETAIL MEAT IN SOUTH ITALY. *Italian Journal of Food Science*. (2020) 32(2):410-9. doi: 10.14674/IJFS-1629

29. Sivakumar M, Dubal ZB, Kumar A, Bhilegaonkar K, Vinodh Kumar OR, Kumar S, et al. Virulent methicillin resistant Staphylococcus aureus (MRSA) in street vended foods. *Journal of Food Science and Technology*. (2019) 56(3):1116-26. doi: 10.1007/s13197-019-03572-5

30. Naas HT, Edarhoby RA, Garbaj AM, Azwai SM, Abolghait SK, Gammoudi FT, et al. Occurrence, characterization, and antibiogram of Staphylococcus aureus in meat, meat products, and some seafood from Libyan retail markets. *Vet World*. (2019) 12(6):925-31. doi: 10.14202/vetworld.2019.925-931

31. El-Ghareeb W, Almathen F, Fayez M, Alsultan R. METHICILLIN RESISTANT STAPHYLOCOCCUS AUREUS (MRSA) IN CAMEL MEAT: PREVALENCE AND ANTIBIOTIC SUSCEPTIBILITY. *Slovenian Veterinary Research*. (2019) 56(22-Suppl). doi: 10.26873/SVR-764-2019

32. Kim YB, Seo KW, Jeon HY, Lim SK, Lee YJ. Characteristics of the antimicrobial resistance of Staphylococcus aureus isolated from chicken meat produced by different integrated broiler operations in Korea. *Poultry Science*. (2018) 97(3):962-9. doi: 10.3382/ps/pex357

33. Thapaliya D, Forshey BM, Kadariya J, Quick MK, Farina S, A OB, et al. Prevalence and molecular characterization of Staphylococcus aureus in commercially available meat over a one-year period in Iowa, USA. *Food Microbiol*. (2017) 65:122-9. doi: 10.1016/j.fm.2017.01.015

34. Osman K, Alvarez-Ordóñez A, Ruiz L, Badr J, ElHofy F, Al-Maary KS, et al. Antimicrobial resistance and virulence characterization of Staphylococcus aureus and coagulase-negative staphylococci from imported beef meat. *Ann Clin Microbiol Antimicrob*. (2017) 16(1):35. doi: 10.1186/s12941-017-0210-4

35. Arslan S, Özdemir F. Molecular characterization and detection of enterotoxins, methicillin resistance genes and antimicrobial resistance of Staphylococcus aureus from fish and ground beef. *Pol J Vet Sci*. (2017) 20(1):85-94. doi: 10.1515/pjvs-2017-0012

36. Zogg AL, Zurfluh K, Nüesch-Inderbinen M, Stephan R. Characteristics of ESBL-producing Enterobacteriaceae and Methicillinresistant Staphylococcus aureus (MRSA) isolated from Swiss and imported raw poultry meat collected at retail level. *Schweiz Arch Tierheilkd*. (2016) 158(6):451-6. doi: 10.17236/sat00071

37. Raji MA, Garaween G, Ehricht R, Monecke S, Shibl AM, Senok A. Genetic Characterization of Staphylococcus aureus Isolated from Retail Meat in Riyadh, Saudi Arabia. *Front Microbiol*. (2016) 7:911. doi: 10.3389/fmicb.2016.00911

38. Rahimi F, Karimi S. Isolation of Methicillin-Resistant Staphylococcus aureus Strains Producing Enterotoxins A, K and Q From Chicken Meat in Isfahan, Iran, 2014. *Arch Clin Infect*. (2016) 11(4):e35601. doi: 10.5812/archcid.35601

39. Sallam KI, Abd-Elghany SM, Elhadidy M, Tamura T. Molecular Characterization and Antimicrobial Resistance Profile of Methicillin-Resistant Staphylococcus aureus in Retail Chicken. *J Food Prot*. (2015) 78(10):1879-84. doi: 10.4315/0362-028x.Jfp-15-150

40. Dhup V, Kearns AM, Pichon B, Foster HA. First report of identification of livestock-associated MRSA ST9 in retail meat in England. *Epidemiol Infect*. (2015) 143(14):2989-92. doi: 10.1017/s0950268815000126

41. Beninati C, Reich F, Muscolino D, Giarratana F, Panebianco A, Klein G, et al. ESBL-Producing Bacteria and MRSA Isolated from Poultry and Turkey Products Imported from Italy. *Czech Journal of Food Sciences*. (2015) 33(2):2015-97. doi: 10.17221/428/2014-CJFS

42. Abdalrahman LS, Stanley A, Wells H, Fakhr MK. Isolation, Virulence, and Antimicrobial Resistance of Methicillin-Resistant Staphylococcus aureus (MRSA) and Methicillin Sensitive Staphylococcus aureus (MSSA) Strains from Oklahoma Retail Poultry Meats. *Int J Environ Res Public Health*. (2015) 12(6):6148-61. doi: 10.3390/ijerph120606148

43. Zarfel G, Galler H, Luxner J, Petternel C, Reinthaler FF, Haas D, et al. Multiresistant bacteria isolated from chicken meat in Austria. *Int J Environ Res Public Health*. (2014) 11(12):12582-93. doi: 10.3390/ijerph111212582

44. Vossenkuhl B, Brandt J, Fetsch A, Käsbohrer A, Kraushaar B, Alt K, et al. Comparison of spa types, SCCmec types and antimicrobial resistance profiles of MRSA isolated from turkeys at farm, slaughter and from retail meat indicates transmission along the production chain. *PLoS One*. (2014) 9(5):e96308. doi: 10.1371/journal.pone.0096308

45. Jackson CR, Davis JA, Barrett JB. Prevalence and characterization of methicillin-resistant Staphylococcus aureus isolates from retail meat and humans in Georgia. *J Clin Microbiol*. (2013) 51(4):1199-207. doi: 10.1128/jcm.03166-12

46. Hu S-k, Liu S-y, Hu W-f, Zheng T-l, Xu J-g. Molecular biological characteristics of Staphylococcus aureus isolated from food. *European Food Research and Technology*. (2013) 236(2):285-91. doi: 10.1007/s00217-012-1887-4

47. O'Brien AM, Hanson BM, Farina SA, Wu JY, Simmering JE, Wardyn SE, et al. MRSA in conventional and alternative retail pork products. *PLoS One*. (2012) 7(1):e30092. doi: 10.1371/journal.pone.0030092

48. Molla B, Byrne M, Abley M, Mathews J, Jackson CR, Fedorka-Cray P, et al. Epidemiology and genotypic characteristics of methicillin-resistant Staphylococcus aureus strains of porcine origin. *J Clin Microbiol*. (2012) 50(11):3687-93. doi: 10.1128/jcm.01971-12

49. Kelman A, Soong YA, Dupuy N, Shafer D, Richbourg W, Johnson K, et al. Antimicrobial susceptibility of Staphylococcus aureus from retail ground meats. *J Food Prot*. (2011) 74(10):1625-9. doi: 10.4315/0362-028x.Jfp-10-571

50. Hanson BM, Dressler AE, Harper AL, Scheibel RP, Wardyn SE, Roberts LK, et al. Prevalence of Staphylococcus aureus and methicillin-resistant Staphylococcus aureus (MRSA) on retail meat in Iowa. *J Infect Public Health*. (2011) 4(4):169-74. doi: 10.1016/j.jiph.2011.06.001

51. Fessler AT, Kadlec K, Hassel M, Hauschild T, Eidam C, Ehricht R, et al. Characterization of methicillin-resistant Staphylococcus aureus isolates from food and food products of poultry origin in Germany. *Appl Environ Microbiol*. (2011) 77(20):7151-7. doi: 10.1128/aem.00561-11

52. Bhargava K, Wang X, Donabedian S, Zervos M, de Rocha L, Zhang Y. Methicillin-resistant Staphylococcus aureus in retail meat, Detroit, Michigan, USA. *Emerg Infect Dis*. (2011) 17(6):1135-7. doi: 10.3201/eid/1706.101905

53. Weese JS, Reid-Smith R, Rousseau J, Avery B. Methicillin-resistant Staphylococcus aureus (MRSA) contamination of retail pork. *Can Vet J*. (2010) 51(7):749-52. doi:

54. Midori H, Fumihiko K, Tetsuya H, Yono S, Norinaga M, Kanji S, et al. Antibiotic Resistance in Bacterial Pathogens from Retail Raw Meats and Food-Producing Animals in Japan. *Journal of Food Protection*. (2012) 75(10):1774-82. doi: 10.4315/0362-028X.JFP-11-479

55. Huber H, Koller S, Giezendanner N, Stephan R, Zweifel C. Prevalence and characteristics of meticillin-resistant Staphylococcus aureus in humans in contact with farm animals, in livestock, and in food of animal origin, Switzerland, 2009. *Euro Surveill*. (2010) 15(16). doi:

56. Pu S, Han F, Ge B. Isolation and characterization of methicillin-resistant Staphylococcus aureus strains from Louisiana retail meats. *Appl Environ Microbiol*. (2009) 75(1):265-7. doi: 10.1128/aem.01110-08

57. van Loo IH, Diederen BM, Savelkoul PH, Woudenberg JH, Roosendaal R, van Belkum A, et al. Methicillin-resistant Staphylococcus aureus in meat products, the Netherlands. *Emerg Infect Dis*. (2007) 13(11):1753-5. doi: 10.3201/eid1311.070358

58. Savariraj WR, Ravindran NB, Kannan P, Paramasivam R, Senthilkumar T, Kumarasamy P, et al. Prevalence, antimicrobial susceptibility and virulence genes of Staphylococcus aureus isolated from pork meat in retail outlets in India. *Journal of Food Safety*. (2019) 39(1):e12589. doi: 10.1111/jfs.12589

59. Rahimi F, Shafiei R. Characteristics of enterotoxin-producing methicillin-resistant Staphylococcus aureus strains isolated from meat in Tehran, Iran. *Journal of Consumer Protection and Food Safety*. (2019) 14(4):389-98. doi: 10.1007/s00003-019-01239-z

60. Velasco V, Vergara JL, Bonilla AM, Muñoz J, Mallea A, Vallejos D, et al. Prevalence and Characterization of Staphylococcus aureus Strains in the Pork Chain Supply in Chile. *Foodborne Pathog Dis*. (2018) 15(5):262-8. doi: 10.1089/fpd.2017.2381

61. El Bayomi RM, Ahmed HA, Awadallah MA, Mohsen RA, Abd El-Ghafar AE, Abdelrahman MA. Occurrence, Virulence Factors, Antimicrobial Resistance, and Genotyping of Staphylococcus aureus Strains Isolated from Chicken Products and Humans. *Vector Borne Zoonotic Dis*. (2016) 16(3):157-64. doi: 10.1089/vbz.2015.1891

62. Hironori T, Serajus S, Debabrata B. Contamination of post-harvest poultry products with multidrug resistant Staphylococcus aureus in Maryland-Washington DC metro area. *Food Control*. (2016) 65:132-5. doi: 10.1016/j.foodcont.2016.01.024

63. Benito D, Gómez P, Lozano C, Estepa V, Gómez-Sanz E, Zarazaga M, et al. Genetic lineages, antimicrobial resistance, and virulence in Staphylococcus aureus of meat samples in Spain: analysis of immune evasion cluster (IEC) genes. *Foodborne Pathog Dis*. (2014) 11(5):354-6. doi: 10.1089/fpd.2013.1689

64. He W, Liu Y, Qi J, Chen H, Zhao C, Zhang F, et al. Food-animal related Staphylococcus aureus multidrug-resistant ST9 strains with toxin genes. *Foodborne Pathog Dis*. (2013) 10(9):782-8. doi: 10.1089/fpd.2012.1452

65. Boost MV, Wong A, Ho J, O'Donoghue M. Isolation of methicillin-resistant Staphylococcus aureus (MRSA) from retail meats in Hong Kong. *Foodborne Pathog Dis*. (2013) 10(8):705-10. doi: 10.1089/fpd.2012.1415

66. Guran HS, Kahya S. Species Diversity and Pheno- and Genotypic Antibiotic Resistance Patterns of Staphylococci Isolated from Retail Ground Meats. *J Food Sci*. (2015) 80(6):M1291-8. doi: 10.1111/1750-3841.12893

67. Petternel C, Galler H, Zarfel G, Luxner J, Haas D, Grisold AJ, et al. Isolation and characterization of multidrug-resistant bacteria from minced meat in Austria. *Food Microbiol*. (2014) 44:41-6. doi: 10.1016/j.fm.2014.04.013

68. Chairat S, Gharsa H, Lozano C, Gómez-Sanz E, Gómez P, Zarazaga M, et al. Characterization of Staphylococcus aureus from Raw Meat Samples in Tunisia: Detection of Clonal Lineage ST398 from the African Continent. *Foodborne Pathog Dis*. (2015) 12(8):686-92. doi: 10.1089/fpd.2015.1958

69. Aklilu E, Nurhardy AD, Mokhtar A, Zahirul IK, Rokiah AS. Molecular detection of methicillin-resistant Staphylococcus aureus (MRSA) and methicillin-resistant Staphylococcus epidermidis (MRSE) isolates in raw chicken meat. *International Food Research Journal*. (2016) 23(1):322-5. . doi:

70. Krumova-Valcheva G, Mateva G, Milanov M, Gyurova E, Daskalov H. DETECTION AND ANTIMICROBIAL RESISTANCE OF COAGULASE- POSITIVE STAPHYLOCOCCUS AUREUS AND METHICILIN RESISTANT STAPHYLOCOCCUS AUREUS ISOLATED FROM RAW PORK IN THE RETAIL. *Tradition and Modernity in Veterinary Medicine*. (2024) 9(1):39-49. doi: 10.5281/zenodo.12705412

71. Koláčková I, Koukalová K, Karpísková R. Prevalence and characteristics of the bacteria Staphylococcus aureus in pig meat. *Epidemiologie Mikrobiologie Imunologie*. (2014) 63(3):191-4. doi:

72. Kanaan MHG, Al-Isawi AJO. Prevalence of methicillin or multiple drug-resistant Staphylococcus aureus in cattle meat marketed in Wasit province. *Tradition and Modernity in Veterinary Medicine*. (2019) 9(1):39-49. doi: 10.35124/bca.2019.19.1.495

73. Zehra A, Gulzar M, Singh R, Kaur S, Gill JPS. Prevalence, multidrug resistance and molecular typing of methicillin-resistant Staphylococcus aureus (MRSA) in retail meat from Punjab, India. *J Glob Antimicrob Resist*. (2019) 16:152-8. doi: 10.1016/j.jgar.2018.10.005

74. Weese JS, Avery BP, Reid-Smith RJ. Detection and quantification of methicillin-resistant Staphylococcus aureus (MRSA) clones in retail meat products. *Lett Appl Microbiol*. (2010) 51(3):338-42. doi: 10.1111/j.1472-765X.2010.02901.x

75. Xin W, Xiaoya T, Xiaodong X, Baowei Y, Meili X, Jianghong M, et al. Staphylococcus aureus and methicillin-resistant Staphylococcus aureus in retail raw chicken in China. *Food Control*. (2013) 29(1):103-6. doi: 10.1016/j.foodcont.2012.06.002

76. Tang Y, Larsen J, Kjeldgaard J, Andersen PS, Skov R, Ingmer H. Methicillin-resistant and -susceptible Staphylococcus aureus from retail meat in Denmark. *Int J Food Microbiol*. (2017) 249:72-6. doi: 10.1016/j.ijfoodmicro.2017.03.001

77. Pauly N, Wichmann-Schauer H, Ballhausen B, Torres Reyes N, Fetsch A, Tenhagen BA. Detection and quantification of methicillin-resistant Staphylococcus aureus in fresh broiler meat at retail in Germany. *Int J Food Microbiol*. (2019) 292:8-12. doi: 10.1016/j.ijfoodmicro.2018.11.025

78. Narvaez-Bravo C, Toufeer M, Weese SJ, Diarra MS, Deckert AE, Reid-Smith R, et al. Prevalence of methicillin-resistant Staphylococcus aureus in Canadian commercial pork processing plants. *J Appl Microbiol*. (2016) 120(3):770-80. doi: 10.1111/jam.13024

79. Mama OM, Morales L, Ruiz-Ripa L, Zarazaga M, Torres C. High prevalence of multidrug resistant S. aureus-CC398 and frequent detection of enterotoxin genes among non-CC398 S. aureus from pig-derived food in Spain. *Int J Food Microbiol*. (2020) 320:108510. doi: 10.1016/j.ijfoodmicro.2020.108510

80. Sun C, Chen B, Hulth A, Schwarz S, Ji X, Nilsson LE, et al. Genomic analysis of Staphylococcus aureus along a pork production chain and in the community, Shandong Province, China. *Int J Antimicrob Agents*. (2019) 54(1):8-15. doi: 10.1016/j.ijantimicag.2019.03.022

81. Lozano C, López M, Gómez-Sanz E, Ruiz-Larrea F, Torres C, Zarazaga M. Detection of methicillin-resistant Staphylococcus aureus ST398 in food samples of animal origin in Spain. *J Antimicrob Chemother*. (2009) 64(6):1325-6. doi: 10.1093/jac/dkp378

82. Liu C-x, Xiao Y-p, Hu D-w, Liu J-x, Chen W, Ren D-x. The safety evaluation of chilled pork from online platform in China. *Food Control*. (2019) 96:244-50. doi: 10.1016/j.foodcont.2018.09.025

83. Li Q, Li Y, Tang Y, Meng C, Ingmer H, Jiao X. Prevalence and characterization of Staphylococcus aureus and Staphylococcus argenteus in chicken from retail markets in China. *Food Control*. (2019) 96:158-64. doi: 10.1016/j.foodcont.2018.08.030

84. Li L, Ye L, Yu L, Zhou C, Meng H. Characterization of Extended Spectrum Β-Lactamase Producing Enterobacteria and Methicillin-Resistant Staphylococcus aureus Isolated from Raw Pork and Cooked Pork Products in South China. *Journal of Food Science*. (2016) 81(7):M1773-M7. doi: <https://doi.org/10.1111/1750-3841.13346>

85. Li H, Tang T, Stegger M, Dalsgaard A, Liu T, Leisner JJ. Characterization of antimicrobial-resistant Staphylococcus aureus from retail foods in Beijing, China. *Food Microbiol*. (2021) 93:103603. doi: 10.1016/j.fm.2020.103603

86. Ge B, Mukherjee S, Hsu CH, Davis JA, Tran TTT, Yang Q, et al. MRSA and multidrug-resistant Staphylococcus aureus in U.S. retail meats, 2010-2011. *Food Microbiol*. (2017) 62:289-97. doi: 10.1016/j.fm.2016.10.029

87. Fox A, Pichon B, Wilkinson H, Doumith M, Hill RL, McLauchlin J, et al. Detection and molecular characterization of Livestock-Associated MRSA in raw meat on retail sale in North West England. *Lett Appl Microbiol*. (2017) 64(3):239-45. doi: 10.1111/lam.12709

88. de Boer E, Zwartkruis-Nahuis JT, Wit B, Huijsdens XW, de Neeling AJ, Bosch T, et al. Prevalence of methicillin-resistant Staphylococcus aureus in meat. *Int J Food Microbiol*. (2009) 134(1-2):52-6. doi: 10.1016/j.ijfoodmicro.2008.12.007

89. Costa WL, Ferreira Jdos S, Carvalho JS, Cerqueira ES, Oliveira LC, Almeida RC. Methicillin-resistant Staphylococcus aureus in raw meats and prepared foods in public hospitals in salvador, Bahia, Brazil. *J Food Sci*. (2015) 80(1):M147-50. doi: 10.1111/1750-3841.12723

90. Cho JI, Joo IS, Choi JH, Jung KH, Choi EJ, Son NR, et al. Distribution of Methicillin-resistant Staphylococcus aureus (MRSA) in RAW meat and fish samples in Korea. *Food Science and Biotechnology*. (2014) 23(3):999-1003. doi: 10.1007/s10068-014-0135-z

91. Buyukcangaz E, Velasco V, Sherwood JS, Stepan RM, Koslofsky RJ, Logue CM. Molecular typing of Staphylococcus aureus and methicillin-resistant S. aureus (MRSA) isolated from animals and retail meat in North Dakota, United States. *Foodborne Pathog Dis*. (2013) 10(7):608-17. doi: 10.1089/fpd.2012.1427

92. Abolghait SK, Fathi AG, Youssef FM, Algammal AM. Methicillin-resistant Staphylococcus aureus (MRSA) isolated from chicken meat and giblets often produces staphylococcal enterotoxin B (SEB) in non-refrigerated raw chicken livers. *Int J Food Microbiol*. (2020) 328:108669. doi: 10.1016/j.ijfoodmicro.2020.108669

93. Ruban SW, Babu RN, Abraham RJJ, Senthilkumar TMA, Kumraswamy P, Rao VA. Prevalence of methicillin resistant Staphylococcus aureus in retail buffalo meat in Chennai, India. *Buffalo Bulletin*. (2018) 37(1):51-8. doi:

94. Saadati A, Mashak Z, Yarmand MS. Prevalence and Molecular Characterization of Enterotoxin- and Antibiotic Resistance-Encoding Genes in the Methicillin-resistant Staphylococcus aureus Recovered From Poultry Meat. *Egyptian Journal of Veterinary Science*. (2021) 52(2):163-73. doi: 10.21608/ejvs.2021.48755.1202

95. Gökmen M. Prevalence, molecular identification and determination of antibiotic susceptibility of methicillin-resistant Staphylococcus aureus in raw meat *Turkish Journal of Veterinary Research*. (2024) 8(1):29-33. doi: 10.47748/tjvr.1381939

96. Lim SK, Nam HM, Park HJ, Lee HS, Choi MJ, Jung SC, et al. Prevalence and characterization of methicillin-resistant Staphylococcus aureus in raw meat in Korea. *J Microbiol Biotechnol*. (2010) 20(4):775-8. doi:

97. Gelbíčová T, Brodíková K, Karpíšková R. Livestock-associated methicillin-resistant Staphylococcus aureus in Czech retailed ready-to-eat meat products. *Int J Food Microbiol*. (2022) 374:109727. doi: 10.1016/j.ijfoodmicro.2022.109727

98. Chan PA, Wakeman SE, Angelone A, Mermel LA. Investigation of multi-drug resistant microbes in retail meats. *Journal of Food Agriculture & Environment*. (2008) 6(3-4):71-5.
